# Qa-SNARE syntaxin 18 mediates lipid droplet fusion with SNAP23 and SEC22B

**DOI:** 10.1038/s41421-023-00613-4

**Published:** 2023-11-21

**Authors:** Yuhui Fu, Binbin Ding, Xiaoxia Liu, Shangang Zhao, Fang Chen, Linsen Li, Yi Zhu, Jingxuan Zhao, Zhen Yuan, Yafeng Shen, Chaofeng Yang, Mengle Shao, She Chen, Perry E. Bickel, Qing Zhong

**Affiliations:** 1 Key Laboratory of Cell Differentiation and Apoptosis of Chinese Ministry of Education, Department of Pathophysiology, Shanghai Jiao Tong University School of Medicine, Shanghai, China; 2 Department of Biochemistry and Molecular Biology, School of Basic Medicine, Tongji Medical College, Huazhong University of Science and Technology, Wuhan, Hubei China; 3 Sam and Ann Barshop Institute for Longevity and Aging Studies, Division of Endocrinology and Diabetes, Department of Medicine, University of Texas Health Science Center at San Antonio, San Antonio, TX USA; 4 Children’s Nutrition Research Center, Department of Pediatrics, Baylor College of Medicine, Houston, TX USA; 5 MedChem Service Unit of Shanghai Haoyuan Chemexpress Co., Ltd., Shanghai, China; 6 Shanghai Institute of Precision of Medicine, Shanghai Ninth People’s Hospital, Shanghai Jiao Tong University School of Medicine, Shanghai, China; 7 Division of Endocrinology, Department of Internal Medicine, University of Texas Southwestern Medical Center, Dallas, TX USA; 8 CAS Key Laboratory of Molecular Virology and Immunology, The Center for Microbes, Development and Health, Shanghai Institute of Immunity and Infection, Chinese Academy of Sciences, Shanghai, China; 9 National Institute of Biological Sciences, Beijing, China

**Keywords:** Membrane fusion, SNARE, Organelles

## Abstract

Lipid droplets (LDs) are dynamic lipid storage organelles that can sense and respond to changes in systemic energy balance. The size and number of LDs are controlled by complex and delicate mechanisms, among which, whether and which SNARE proteins mediate LD fusion, and the mechanisms governing this process remain poorly understood. Here we identified a SNARE complex, syntaxin 18 (STX18)–SNAP23–SEC22B, that is recruited to LDs to mediate LD fusion. STX18 targets LDs with its transmembrane domain spanning the phospholipid monolayer twice. STX18–SNAP23–SEC22B complex drives LD fusion in adiposome lipid mixing and content mixing in vitro assays. CIDEC/FSP27 directly binds STX18, SEC22B, and SNAP23, and promotes the lipid mixing of SNAREs-reconstituted adiposomes by promoting LD clustering. Knockdown of STX18 in mouse liver via AAV resulted in smaller liver and reduced LD size under high-fat diet conditions. All these results demonstrate a critical role of the SNARE complex STX18–SNAP23–SEC22B in LD fusion.

## Introduction

Lipid droplets (LDs) are ubiquitous organelles in eukaryotic cells. As cellular energy hubs, they play an important role in the maintenance of cellular homeostasis and their malfunction is highly related to fatty liver, liver fibrosis, diabetes, obesity, and other metabolic diseases^1–3^. Fatty acids as energy source for cells, are stored as triglycerol (TG) in LDs, as excess free fatty acids are toxic for the cells. Upon starvation, the fatty acids stored as TG are mobilized by lipolysis and released from LDs to provide energy, which leads to dynamic changes in the size, number, distribution, and mobility of LDs.

All LDs have very similar structures, but are unique from most other organelles wrapped by a phospholipid bilayer. LDs consist of a hydrophobic core of TG and sterol esters, which are enclosed by a phospholipid monolayer, with the hydrophilic phosphate ester head facing the cytoplasm, and the hydrophobic chain fatty acid tail facing the core^4^. There are a variety of proteins decorating the surface of LDs. They are attached to or inserted into the phospholipid monolayer through hydrophobic hairpins, amphipathic helices, or fatty acid modifications. The LD proteome is tightly regulated and closely related to the dynamics and functions of LDs^5,6^.

LDs are highly dynamic organelles, alternating between periods of growth and consumption. The biogenesis of LDs starts from the endoplasmic reticulum (ER) membrane, including the following steps: (1) neutral lipid synthesis and oil len formation; (2) LD budding from ER; (3) LD growth and maturation. LDs can expand through droplet–droplet fusion/coalescence, transfer of TG from ER or adjacent LDs, or TG synthesis on the LD surface^7^.

The fusion/coalescence of LDs facilitates the storage efficiency of TG. It also reduces the surface area of LDs and the risk of TG degradation by lipases. The cell death-inducing DFFA-like effector (CIDE) family of proteins, including CIDEA, CIDEB, and CIDEC (also known as FSP27), were reported to be enriched at LD–LD contact sites to mediate lipid transfer between LDs^8^. Homozygous mutation in the CIDEC/FSP27 has been found in human patients with fatty liver, hypertension, and insulin-resistant diabetes^9^. CIDEC/FSP27 can form stable trans-organelle oligomer to facilitate lipid transfer from small LDs to large ones in adipocytes^8,10^. Especially, for CIDEC/FSP27, its gel-like condensation at LD–LD contact sites generates lipid-permeable plates for LD fusion^11^. However, so far reconstitution experiments with in vitro synthesized LDs and recombinant full-length CIDEC/FSP27 were still lacking and the detailed mechanism of how lipid-permeable plates were formed due to the condensation of CIDEC/FSP27 is unknown.

The process of membrane fusion is highly conserved in evolution. According to the classic theory, membrane fusion is driven by the zipping of SNAREs (soluble N-ethylmaleimide sensitive factor attachment protein receptors) into a four-helix bundle with the help of other proteins, such as Rab GTPase, SM proteins (Sec1/SM family proteins), tethering proteins and others^12^. SNAREs are membrane-anchored proteins via transmembrane domains (TMDs) or post-translationally modified hydrophobic tails (palmitoylation, etc.). All SNARE proteins contain evolutionarily conserved coiled-coil SNARE motifs. According to the amino acid that is present in the zero ionic layer of the SNARE domain, SNARE proteins are divided into R-SNAREs (with arginine in the zero ionic layer) and Q-SNAREs (with glutamine in the zero ionic layer)^13^. There are three Q-SNARE families: Qa, Qb and Qc. Usually, R-, Qa-, Qb-, and Qc-SNAREs form a four-helix bundle by spontaneously zippering from the N-terminus to the C-terminus of the SNARE domains. During this process, the *trans*-SNARE complex pulls and distorts the two membranes on which the proteins are anchored. With the help of other proteins, the membrane lipids are reorganized, and the membranes are eventually fused.

Despite being a core machinery of membrane fusion, whether SNAREs mediate LD fusion is not clear. Previously, it was reported that at the contact site between LDs and ER, ER-resident SNARE complex (syntaxin 18 (STX18)–USE1–BNIP1) can function together with NRZ complex (NAG–RINT1–ZW10) and Rab18 as a bridging complex to maintain this contact site^14^. In another study, it was reported that SNARE complex (STX5–SNAP23–VAMP4) can drive LD fusion^15^, but this hypothesis was under debate as no in vitro fusion evidence directly supports it. Additionally, SNAP23 was also reported to be involved in LD formation^16^. Recently, it was reported that SNAREs are involved in protein targeting from ER to LD, probably through a fused membrane bridge between LD and ER, but whether SNAREs mediate the LD–ER fusion is not demonstrated^17^. Besides, how SNARE proteins with TMD are located on phospholipid monolayer is elusive. Whether SNAREs are critical fusion machinery for LD fusion remains to be an important fundamental question to be answered.

In this work, we identified a new set of SNAREs, STX18–SNAP23–SEC22B, that mediates LD fusion. They are located on LDs and form a stable SNARE complex both in vivo and in vitro. The TMD of STX18 spans the phospholipid monolayer twice, with its C-terminal amino acids facing the cytosol. Both the FRET-based lipid mixing assay and content mixing assay indicate that STX18–SNAP23–SEC22B mediates the fusion between LDs. Besides, CIDEC/FSP27 interacts directly with STX18, SEC22B, and SNAP23, promoting LD fusion driven by SNAREs. Furthermore, mouse liver with STX18 knockdown shows smaller LDs upon high-fat diet (HFD) feeding. Altogether, we demonstrate STX18–SNAP23–SEC22B as a key SNARE complex mediating LD fusion.

## Results

### STX18, SNAP23, and SEC22B mediate LD fusion in cells

**Fig. 1:**
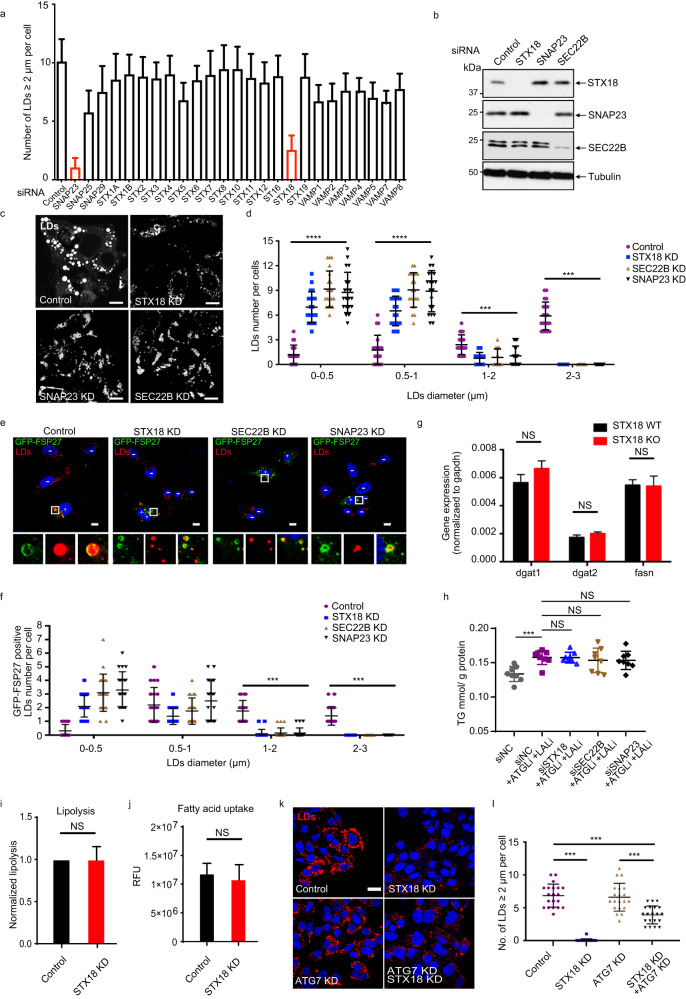
Identification of SNAREs STX18, SNAP23, SEC22B for LD size control. **a** HepG2 cells were transfected with control siRNA or siRNAs targeting SNARE genes for 48 h and then treated with 0.2 mM oleic acid-modified bovine serum albumin (OA-BSA) for 12 h. LDs were stained by BODIPY 558/568 C_12_ for additional 1 h before fixing the cells. The graph shows the quantification of large LDs (> 2 µm) by analyzing the average LD number in 50 cells. **b** HepG2 cells were transfected with control, *STX18*, *SEC22B* or *SNAP23* siRNAs for 48 h and then cells were analyzed via western blot. **c** HepG2 cells were transfected with control, *STX18*, *SEC22B* or *SNAP23* siRNAs for 36 h and then treated with 0.2 mM OA-BSA for 12 h. LDs were stained by BODIPY 558/568 C_12_ for additional 1 h before cell fixing. Cells were analyzed via fluorescence. Scale bars, 10 μm. **d** Quantification of LDs with different sizes per cell (> 50 cells) in **c**. Student’s *t*-test; ***P < 0.001, ****P < 0.0001. **e** U_2_OS cells were transfected with control, *STX18*, *SNAP23* or *SEC22B* siRNA for 24 h, and then cells were further transfected with GFP-FSP27 for another 24 h. LDs were stained by BODIPY 558/568 C_12_ for additional 1 h before cell fixing. “+” labeling in cells indicates overexpression of GFP-Fsp27; “–” labeling in cells indicates no expression of GFP-Fsp27. Scale bars, 10 μm (main images). **f** Quantification of GFP-FSP27-positive LD number per cell (> 50 cells). Student’s *t*-test; ***P < 0.001. **g** The mRNA levels of *dgat1*, *dgat2*, and *fasn* in STX18 WT or KO HepG2 cells were analyzed via RT-qPCR. Student’s *t*-test; NS, not significant. **h** TG content in HepG2 cells. Cells were transfected with siNC, siSTX18, siSNAP23 or siSEC22B. After 24 h, 30 μM ATGLi plus 50 μM LALi or DMSO was added into the cells. 12 h later, 0.2 mM OA-BSA was added into the cells and kept for another 12 h. The concentration of TG was measured by the commercial kit, and then normalized to protein amount. Unpaired *t*-test; NS, not significant; ***P < 0.001. **i** The neutral lipolysis levels were analyzed in STX18 WT or KD HepG2 cells. Cells were transfected with siNC or siSTX18 for 45 h. After that, the cells were treated with isoproterenol for 3 h, followed by measurement of free glycerol released into the culture medium. NS not significant. Paired *t*-test. **j** The fatty acid uptake levels were analyzed in STX18 WT or KD HepG2 cells. Cells were transfected with siNC or siSTX18 for 48 h, followed by fatty acid uptake measurement. NS not significant. Unpaired *t*-test. **k** ATG7 WT or KD HepG2 cells were transfected with control or STX18 siRNA for 36 h and then treated with 0.2 mM OA-BSA for 12 h. LDs were stained by BODIPY 558/568 C_12_ for additional 1 h before fixing the cells, and cells were analyzed via fluorescence. Scale bar, 10 μm. **l** Quantification of large LDs (≥ 2 µm) per cell (> 50 cells) in **k**. Student’s *t*-test; ***P < 0.001.

Distinct sets of SNARE complexes mediate different types of membrane fusion^18–24^. To identify the specific set of SNARE proteins that mediates LD fusion in liver cells, we utilized siRNA screening to knock down various SNAREs in HepG2 cells. We found that compared to the knockdown of other SNAREs, the knockdown of STX18 or SNAP23 significantly reduced the population of LDs bigger than 2 μm (Fig. 1a, b). Interestingly, STX5 and VAMP4, which were reported to mediate LD fusion in a previous study^15^, showed minimal effects on the population of large LDs (> 2 μm) in our siRNA screening performed in HepG2 cells (Fig. 1a; Supplementary Fig. S1a). BNIP1 and USE1 have also been reported to interact with STX18 in the tethering complex between the ER and LDs in 3T3-L1 cells^14^; however, knockdown of BNIP1 or USE1 (Supplementary Fig. S1b) did not result in significant changes in the number of small LDs (0–0.5 μm and 0.5–1 μm), medium LDs (1–2 μm) and large LDs (> 2 μm) in HepG2 cells treated with oleic acid (OA) (Supplementary Fig. S1c, d), suggesting that STX18 might interact with a new set of SNAREs to mediate LD fusion.

To identify which R-SNARE forms a complex with STX18 (Qa-SNARE) and SNAP23 (Qbc-SNARE), we performed immunoprecipitation-mass spectrometry (IP-MS) of Flag-STX18 to search for its binding R-SNARE. According to the MS results, SEC22B (R-SNARE) and SNAP23 (Qbc-SNARE) were two of the top candidates as STX18 (Qa-SNARE) binding proteins (Supplementary Fig. S1e). Besides, the reverse tandem affinity purification-mass spectrometry (TAP-MS) of ZZ-Flag-SEC22B also indicated interaction among these SNAREs and their possibility to be a SNARE complex (Supplementary Fig. S1f). Furthermore, we verified the effect of STX18, SNAP23, and SEC22B depletion on LD size distribution in a histogram assay. Knockdown of STX18, SNAP23, or SEC22B by siRNAs significantly reduced the size of LDs upon OA treatment, as the population of small LDs increased while the population of large LDs decreased (Fig. 1b–d).

In addition to OA treatment, we also examined whether the depletion of these SNAREs affects the LD growth induced by different mechanisms. Previous study has indicated that overexpression of CIDEC/FSP27 promotes LD growth by facilitating lipid transfer between LDs^8^. We examined whether the depletion of these SNAREs would affect LD growth in this situation. As expected, the size of LDs in CIDEC/FSP27-expressing cells was big, ~0.5–3 μm, while much fewer LDs of ~1–3 μm were observed upon knockdown of STX18, SNAP23, or SEC22B even with overexpression of CIDEC/FSP27 (Fig. 1e, f). Altogether, these data suggest that STX18, SEC22B, and SNAP23 play an important role in LD size control.

LD size is not only influenced by LD fusion, but also likely by many other factors, including LD biosynthesis, growth and turnover, neutral lipolysis in neutral cytoplasm, and LD engulfment in acidic lysosomes. Next, we investigated whether the knockdown of STX18 reduces the LD size by affecting lipid biogenesis, lipolysis, and fatty acid uptake in HepG2 cells. We found that depletion of STX18 had no effect on mRNA levels of crucial genes involved in fatty acid synthesis (*fasn*) and LD biogenesis (*dgat1*, *dgat2*) (Fig. 1g; Supplementary Fig. S1g). Besides, we also measured the TG synthesis levels upon the depletion of STX18, SNAP23, or SEC22B in HepG2 cells. TG content is a reflection of both TG synthesis and lipolysis. To evaluate TG synthesis, we treated cells with both neutral lipase inhibitor (ATGLi) and lysosomal lipase inhibitor (LALi) to block TG degradation. TG levels were increased in siNC-treated HepG2 cells after the addition of ATGLi and LALi as expected, but knockdown of STX18, SNAP23, or SEC22B could not cause further changes in TG levels (Fig. 1h), suggesting that the TG synthesis levels remained unaltered when any of these SNARE proteins were depleted. These results indicate that the decreased large-sized LD population upon STX18, SNAP23, or SEC22B depletion, was likely caused by defects in LD fusion rather than decreased TG synthesis. Moreover, in a prior study by Xu et al.^14^, a decrease in TG levels was observed upon STX18 depletion in 3T3-L1 cells without inhibiting lipase activity. This finding supports the essential role of STX18 in lipid metabolism, which may depend on distinct cellular contexts and genetic backgrounds.

Moreover, neither neutral lipolysis (Fig. 1i) nor fatty acid uptake (Fig. 1j) was affected upon STX18 depletion. These results suggest that the knockdown of STX18 has little if any effect on lipid synthesis, neutral lipolysis, and fatty acid uptake. Furthermore, to exclude the possibility that small LDs are caused by autophagosomal engulfment of large LDs, we depleted the autophagy essential gene ATG7 in HepG2 cells. The inhibition of autophagy by ATG7 siRNA knockdown in STX18 KD HepG2 cells also showed decreased number of large LDs (> 2 μm), indicating that small LDs did not come from the degradation of large ones by autophagy (Fig. 1k, l).

Next, as the biogenesis of LDs starts from the ER membrane, we determined whether ER stress is activated in STX18-depleted cells. Our data showed that knockdown and knockout of STX18 in HepG2 cells failed to induce ER stress as evidenced by lack of induction of CHOP and Xbp1s expression, respectively (Supplementary Fig. S1h–j). Next, we examined the ER structure upon STX18 knockdown in HeLa cells by investigating the distribution and morphology of two ER markers, ER exit site marker Sec31A and ER membrane protein Bap31. The distribution and morphology of both ER proteins displayed no obvious difference between wild-type (WT) and STX18 KD cells (Supplementary Fig. S1k, l). Furthermore, as the perturbed ER homeostasis might change the synthesis of LDs, we examined the synthesis of nascent LDs by LiveDrop probe upon STX18 depletion. LiveDrop is a widely used probe to label nascent LDs^25^. The results showed that neither the area nor the number of LiveDrop puncta changed significantly in STX18 KD HepG2 cells (Supplementary Fig. S1m–o). Therefore, the depletion of STX18 did not change the synthesis of nascent LDs. All these results suggest that the reduction in LD size upon depletion of STX18 is not due to change in ER homeostasis.

According to the above results, we presumed that STX18, SEC22B, and SNAP23 play an important role in LD size control likely through LD fusion.

### STX18, SNAP23, and SEC22B form a SNARE complex

**Fig. 2:**
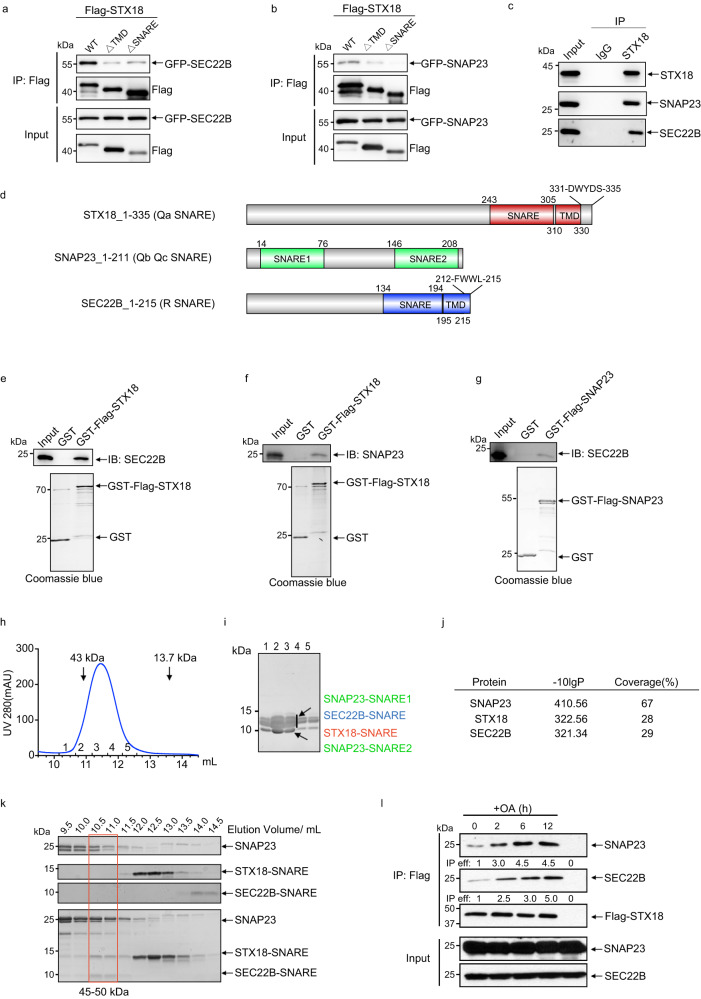
STX18, SNAP23, and SEC22B form a SNARE complex. **a** HEK293T cells transfected with GFP-SEC22B and different Flag-STX18 constructs (Flag-STX18 WT, STX18 ΔTMD, or STX18 ΔSNARE) for 36 h, and then cells were subjected to Flag IP followed by western blot analysis. **b** HEK293T cells transfected with GFP-SNAP23 and different Flag-STX18 constructs (Flag-STX18 WT, STX18 ΔTMD, or STX18 ΔSNARE) for 36 h, and then cells were subjected to Flag IP and analyzed via western blot. **c** Interaction between STX18 and SNAP23, SEC22B verified by endogenous IP experiment in HepG2 cells. **d** Boundaries of different domains in STX18 (red), SNAP23 (green), and SEC22B (blue). The primary sequences of the C-termini of STX18 (331-DWYDS-335) and SEC22B (212-FWWL-215) were indicated. **e** Interaction between purified SEC22B and GST-Flag-tagged STX18 using an in vitro GST pull-down assay followed by immunoblot (IB) and Coomassie blue staining. **f** Interaction between purified SNAP23 and GST-Flag-tagged STX18 using an in vitro GST pull-down assay followed by IB and Coomassie blue staining. **g** Interaction between purified SEC22B and GST-Flag-tagged SNAP23 using an in vitro GST pull-down assay followed by IB and Coomassie blue staining. **h** Purification of mini-SNARE complex with His-SEC22B-SNARE, STX18-SNARE, His-SNAP23-SNARE1, and SNAP23-SNARE2 from *E. coli*. The His-tag affinity purification protein mixture was subjected to Superdex75 increase 10/300 GL and eluted as a single peak. **i** The elution fraction of purified mini-SNARE complex in **h** was assessed by SDS-PAGE. **j** The mini-SNARE complex bands excised from SDS-PAGE in **h** were assessed by MS. **k** The purified SNAP23, STX18-SNARE, SEC22B-SNARE or their in vitro incubated mixture were subjected to Superdex75 10/300 GL, followed by SDS-PAGE analysis. **l** Huh7 cells were transfected with Flag-STX18 for 36 h, treated with or without 0.2 mM OA-BSA for another 2 h, 6 h, 12 h, and then subjected to Flag IP followed by western blot analysis.

It is well known that Qa-, Qb-, Qc- and R-SNAREs form a four-helix bundle to drive membrane fusion; therefore, we next sought to determine whether STX18, SNAP23, and SEC22B form a SNARE complex both in vivo and in vitro. Both IP-MS of Flag-STX18 (Supplementary Fig. S1e) and TAP-MS of ZZ-Flag-tagged SEC22B (Supplementary Fig. S1f) indicated the interaction among STX18, SNAP23 and SEC22B. We first verified their interaction in a co-immunoprecipitation (co-IP) experiment, and the western blot results showed that overexpressed Flag-STX18 co-immunoprecipitated with overexpressed SEC22B and SNAP23 (Fig. 2a, b). Besides, we performed the endogenous co-IP without overexpression of these SNAREs, and the results showed that in HepG2 cells, the endogenous STX18 interacted with endogenous SNAP23 and endogenous SEC22B (Fig. 2c). As STX18 is composed of one TMD and one SNARE domain^26^ (Fig. 2d), we next performed deletion mapping to determine which domains of STX18 are required for its binding with SEC22B and SNAP23 in a co-IP assay. STX18 mutants with deleted SNARE domain significantly reduced its interaction with SNAP23 or SEC22B (Fig. 2a, b), suggesting that SNARE domain interaction is required for the assembly of SNAREs in cells. Besides, deletion of TMD also reduced the interaction between STX18 and SEC22B or SNAP23 (Fig. 2a, b), which is probably due to its important role in the membrane localization of STX18. Furthermore, to verify the direct binding among STX18, SEC22B, and SNAP23, we performed pull-down assays with purified SNARE proteins. GST-STX18 directly bound SEC22B (Fig. 2e) and SNAP23 (Fig. 2f). Meanwhile, GST-SNAP23 interacted directly with SEC22B (Fig. 2g). Next we used more rigorous assays to evaluate the assembly and stoichiometry of the STX18–SNAP23–SEC22B SNAREpin complex. We co-expressed and co-purified four SNARE domains of STX18 (243–305), SEC22B (134–194), and SNAP23 (14–76 and 146–208) in a mini-SNAREpin complex. The size exclusion chromatography (SEC) showed that the co-purified proteins were eluted as a compact complex with molecular weight close to 43 kDa, which was about the sum of the molecular weight of these four SNARE domains, 40 kDa (Fig. 2h, i). The MS results of the purified complex also verified the presence of these four SNARE domains, and the coverage also fits the SNARE length in the whole primary sequence (Fig. 2j). We also tested whether the individually purified SNAREs assemble into a complex in vitro. When expressed individually, not every SNARE domain polypeptide is soluble, we have attempted different fragments and combinations. In the best combination, we used full-length SNAP23 instead of its SNARE domains. The SEC was applied to examine the assembly of STX18–SNARE (243–305), SEC22B–SNARE (134–194) and SNAP23. The SDS-PAGE of SEC fractions showed that at least a portion of STX18–SNARE and most of SEC22B–SNARE changed their elution volume and co-migrate with SNAP23 to a fraction with high molecular weight (Fig. 2k). These results demonstrate that STX18, SNAP23, SEC22B are capable of assembling into a SNAREpin complex in vitro. Furthermore, we tested whether the assembly of this SNARE complex can respond to rapid TG biogenesis induced by OA treatment. The IP results showed that the interaction among STX18, SNAP23, and SEC22B can be obviously observed after OA treatment for 2 h and enhanced with time (Fig. 2l). Taken together, these data suggest that STX18, SNAP23, and SEC22B assemble into a SNARE complex, in response to LD biogenesis induced by OA treatment.

### Targeting of SNAREs to LDs

**Fig. 3:**
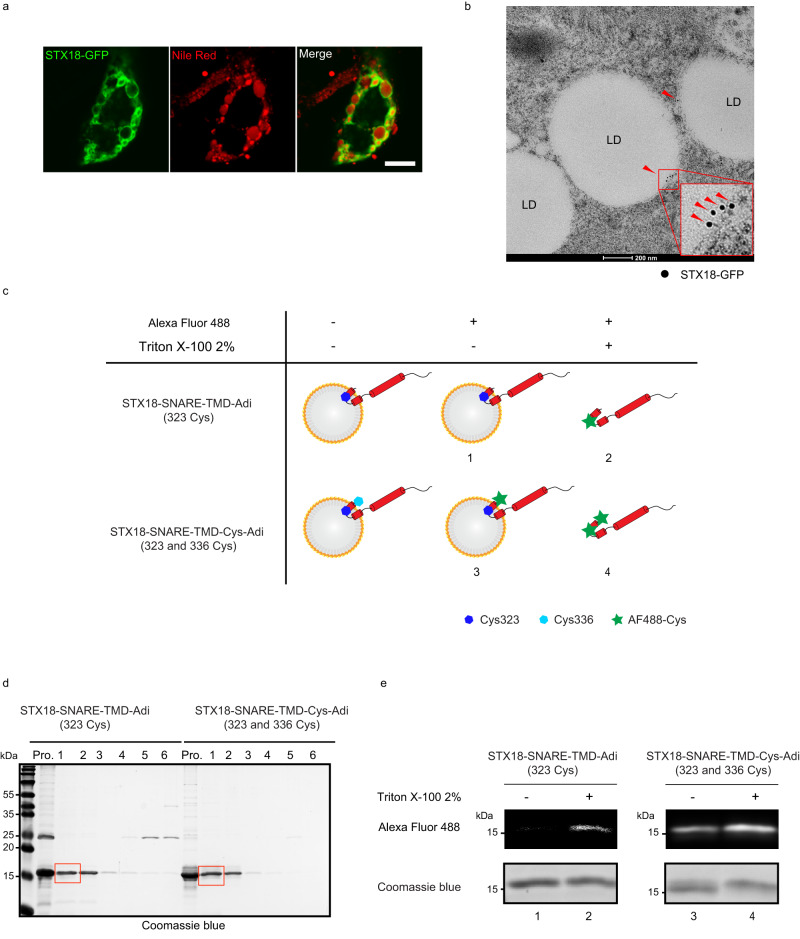
Targeting of SNAREs to LDs. **a** HepG2 cells were transfected with STX18-GFP and then treated with 0.2 mM OA-BSA for 6 h. LDs were stained by Nile Red for 1 h after cell fixing. Cells were analyzed via fluorescence. Scale bar, 10 μm. **b** HepG2 cells transiently expressing STX18-GFP were treated with 0.2 mM OA-BSA for 12 h, and cells were analyzed via immunogold electron microscopy. The anti-GFP primary antibody and gold-conjugated secondary antibody were used to detect GFP signals. Red arrowheads indicate STX18-GFP. Scale bar, 200 nm. **c** Cysteine accessibility of STX18 with predicted orientations on adiposomes, where the TMD spans phospholipid monolayer twice. The cartoon showed that the buried inherent Cys323 (blue hexagon) was not labeled by Alexa Fluor 488 C5 maleimide dye but the exposed artificially engineered Cys336 (cyan hexagon) can be labeled by Alexa Fluor 488 C5 maleimide dye. However, the treatment of 2% Triton X-100 made both Cysteine residues exposed and labeled by Alexa Fluor 488 C5 maleimide dye. Successful labeling of cysteine by AF488 was indicated by green stars. **d** Purified STX18-SNARE-TMD (Cys323) and STX18-SNARE-TMD-Cys (Cys323, Cys336) were reconstituted on adiposomes followed by co-flotation analysis. Fractions were analyzed by SDS-PAGE and Coomassie blue staining. **e** The reconstituted STX18-SNARE-TMD (Cys323) and STX18-SNARE-TMD-Cys (Cys323, Cys336) were labeled by Alexa Fluor 488 C5 maleimide dye with or without 2% Triton X-100. Samples were separated by SDA-PAGE and analyzed by fluorescent imaging (top) and Coomassie blue staining (bottom). The numbers labeled under each sample are corresponding to the numbers in **c**.

We wondered whether STX18 executes its potential fusogenic activity on the surface of LDs. The localization of STX18 on LDs was visualized by confocal microscopy analysis. Upon OA treatment in HepG2 cells, GFP-tagged STX18 co-localized with LDs stained by Nile Red (Fig. 3a), and GFP-tagged SEC22B and SNAP23 also co-localized with LDs labeled by perilipin 2 (Plin2), a specific LD-associated protein (Supplementary Fig. S2a, b). Using electron microscope, we found that GFP-labeled STX18 decorated on the membrane of LDs upon OA treatment (Fig. 3b).

It is well-accepted that LDs comprise a hydrophobic core of TG and sterol esters, enveloped by a phospholipid monolayer. Notably, sequence analysis reveals that SEC22B’s C-terminal region terminates with hydrophobic amino acids (212-FWWL-215) at the end of its TMD (Fig. 2d), fitting well with the hydrophobic LD environment. In contrast, STX18 has several hydrophilic amino acids (331-DWYDS-335) following its TMD (Fig. 2d), the topology of which is the next question we aimed to address.

To figure out the topology of STX18 on phospholipid monolayer of LDs, we first predicted the structure of TMD in STX18 by I-TASSER^27^. The results showed that its TMD can be one intact α-helix or two split short α-helices with a linker in between (Supplementary Fig. S2c). The length of both split α-helices is enough to span the phospholipid monolayer. To verify whether the TMD spans the phospholipid monolayer and to figure out how it spans the phospholipid monolayer, we designed a cysteine accessibility assay with adiposome (artificial LD) associated STX18 (Fig. 3c). The cysteine residues can be specifically labeled with fluorescence through reaction of their thiol groups with electrophile maleimide-linked fluorescence reagents if the cysteines are exposed but not buried in the membrane or the hydrophobic core of adiposomes.

Adiposomes are biochemically reconstituted structures with neutral lipid core covered by phospholipid monolayer membrane, resembling native LDs in terms of structure and composition^28^. We constructed adiposomes following previously published method^28^. Initially, we assessed the size of the adiposomes using Dynamic Light Scattering (DLS), revealing a diameter distribution centered around 180 nm (Supplementary Fig. S2d). Subsequently, we examined the freshly prepared adiposomes using both ultrathin transmission electron microscopy (TEM) and cryo-electron microscopy (cryo-EM) at lower magnifications. Both TEM (Supplementary Fig. S2e, f) and cryo-EM (Supplementary Fig. S2g, h) consistently demonstrated that the average size of the adiposomes was ~160 nm. It is worth noting that the quality and uniformity of these artificially constructed LDs closely resembled those previously reported in the literature^28^.

As STX18-SNARE-TMD (a.a. 243–335) contains only one cysteine residue at the second predicted short α-helix in the TMD (Cys323) that could be labeled with fluorescence, to facilitate mapping, we also generated a mutant with an additional cysteine residue (Cys336) at the extreme C-terminus (Fig. 3c). If the TMD would not span the phospholipid monolayer, both fragments should be labeled with fluorescence dye. If the TMD only spans the phospholipid monolayer once, both cysteines should be buried and neither of them could be labeled. If the TMD spans the phospholipid monolayer twice with both N-terminus and C-terminus of STX18 facing cytosol, Cys323 would be buried and Cys336 would be exposed and labeled with fluorescence (Fig. 3c).

STX18 Cys336 mutation imposes no effect on its secondary structure (Supplementary Fig. S2i). We then reconstituted these two STX18 fragments, STX18-SNARE-TMD and STX18-SNARE-TMD-Cys336 on adispomes, respectively, and their adiposome targeting was confirmed by co-flotation assay (Fig. 3d). The top layers containing the STX18-reconstituted adiposomes were collected (Fig. 3d, red box), and then subjected to the reaction with maleimide-linked Alexa Fluor 488 in the presence or absence of 2% Triton X-100. We verified the labeling efficiency by treating adiposomes with 2% Triton which can lyse adiposomes completely. Both STX18 fragments were labeled with Alexa Fluor 488 in the presence of 2% Triton X-100 (lanes 2 and 4 in Fig. 3e), indicating that the labeling reactions worked well. With the intact adipososmes in the absence of detergent, only STX18-SNARE-TMD-Cys (323 and 336 Cys) fragment was labeled (lane 3 in Fig. 3e), whereas STX18-SNARE-TMD was not labeled (lane 1 in Fig. 3e). These results support the model that TMD of STX18 spans the phospholipid monolayer twice, with its C-terminal tail exposed to the outside of adiposomes and the interval cysteine was buried in the membrane (Fig. 3c). This model also explains the topology of two hydrophilic residues (D334 and S335) at the C-terminal end of STX18. Collectively, these results indicate that STX18, SEC22B, and SNAP23 localize on LDs, and the TMD of STX18 spans the phospholipid monolayer twice.

### STX18–SNAP23–SEC22B mediates lipid mixing and content mixing between adiposomes

**Fig. 4:**
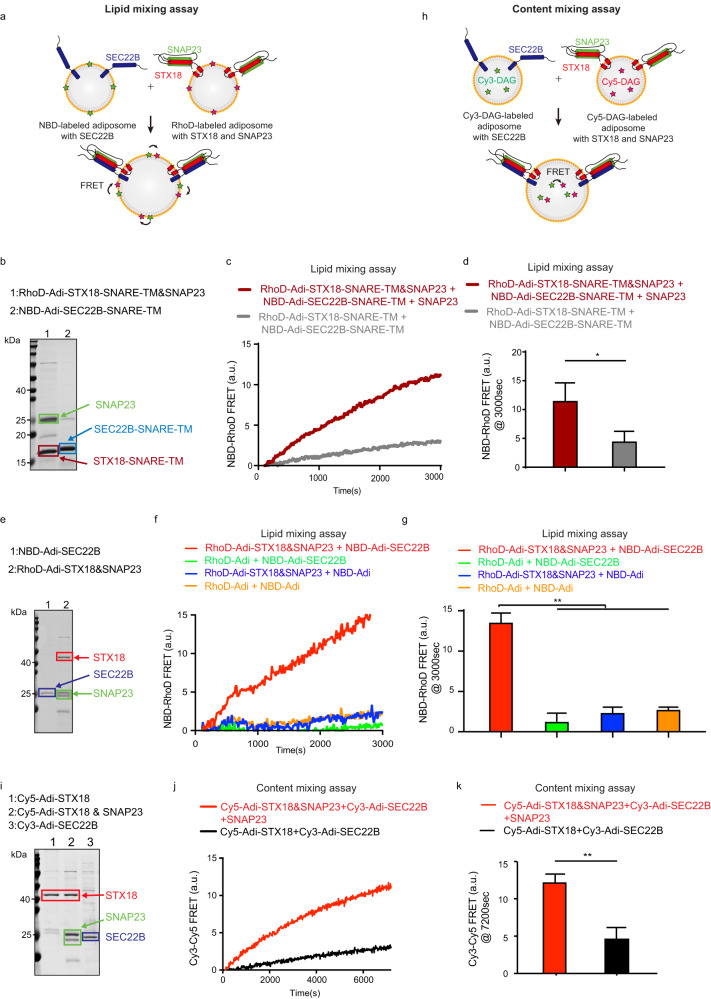
STX18–SNAP23–SEC22B mediated adiposome fusion. **a** Scheme of FRET-based adiposome lipid mixing assay. **b** SDS-PAGE analysis of Rhod-labeled adiposome reconstituted with STX18-SNARE-TM and SNAP23, and NBD-labeled adiposome reconstituted with SEC22B-SNARE-TM, followed by Coomassie blue staining. **c** Lipid mixing between adiposomes reconstituted with the indicated SNAREs was measured from the development of FRET between NBD-PE and Rhod-PE. The NBD-adiposomes were reconstituted with SEC22B-SNARE-TM, while the RhoD-adiposomes were reconstituted with STX18-SNARE-TM and SNAP23. In the complete reaction set, the two adiposomes were mixed with extra SNAP23. For the incomplete reaction set, RhoD-Adi-STX18-SNARE-TM was used to replace RhoD-Adi-STX18-SNARE-TM&SNAP23. No extra SNAP23 was included in the incomplete reaction set. Both reactions were recorded at 37 °C. **d** Quantification of lipid mixing experiments of **c**. Bars represent averages of the FRET signal observed after 3000 s in three independent experiments. Error bars represent SDs. Paired *t*-test; *P < 0.05. **e** SDS-PAGE analysis of Rhod-labeled adiposome reconstituted with STX18 and SNAP23, and NBD-labeled adiposome reconstituted with SEC22B, followed by Coomassie blue staining. **f** Lipid mixing between adiposomes reconstituted with the indicated SNAREs was measured from the development of FRET between NBD-PE and Rhod-PE. The NBD-adiposomes were reconstituted with or without SEC22B, while the RhoD-adiposomes were reconstituted with or without STX18 and SNAP23. The reactions were recorded at 37 °C. **g** Quantification of lipid mixing experiments of **f**. Bars represent averages of the FRET signal observed after 3000 s in three independent experiments. Error bars represent SDs. Unpaired *t*-test; **P < 0.01. **h** Scheme of FRET-based adiposome content mixing assay. **i** SDS-PAGE analysis of reconstituted STX18-adipsomes encapsulated with Cy5-DAG, STX18–SNAP23-adiposomes encapsulated with Cy5-DAG, and SEC22B-adiposomes encapsulated with Cy3-DAG, followed by Coomassie blue staining. **j** Content mixing between adiposomes reconstituted with the indicated SNAREs was measured from the development of FRET between Cy3-DAG and Cy5-DAG. Both STX18-adipsomes and STX18–SNAP23-adiposomes were encapsulated with Cy5-DAG, while SEC22B-adiposomes were encapsulated with Cy3-DAG. In the complete reaction set, extra SNAP23 was added into the reaction. The reactions were recorded at 37 °C. **k** Quantification of content mixing experiments of **j**. Bars represent averages of the FRET signal observed after 7200 s in three independent experiments. Error bars represent SDs. Unpaired *t*-test; **P < 0.01.

To obtain direct evidence that the STX18–SNAP23–SEC22B complex mediates LD fusion, we first performed the SNARE-dependent adiposome lipid mixing assay in vitro (Fig. 4a). We reconstituted NBD-labeled adiposome with SEC22B-SNARE-TMD (134–215) and Rhodamine (Rhod)-labeled adiposome with STX18-SNARE-TMD (243–335) and SNAP23. The SDS-PAGE analysis indicated the successful incorporation of these proteins into adiposomes (Fig. 4b). The cryo-EM imaging displayed the intact morphology of fluorescence-labeled adiposomes as a sphere-shaped structure with a single electron-dense line around (Supplementary Fig. S3a)^29^. Lipid mixing was measured as the fluorescence emission (580 nm) of Rhod acceptor dyes arising from FRET upon excitation of NBD dyes with 460 nm light. The results revealed that STX18-SNARE-TMD, SNAP23, and SEC22B-SNARE-TMD together drove the lipid mixing between adiposomes, but without SNAP23, this lipid mixing was greatly decreased (Fig. 4c, d). Besides, both cryo-EM (Supplementary Fig. S3a, b) and DLS (Fig. S3c) showed that adiposome size was increased after mixing the SNAREs-reconstituted adiposomes together, which is in accordance with their roles in LD fusion. Furthermore, we also examined the lipid mixing between adiposomes decorated by full-length STX18, SNAP23, and SEC22B. The SDS-PAGE analysis indicated that these proteins were incorporated into adiposomes (Fig. 4e). The protein-to-lipid ratios were measured: STX18:phospholipid is ~1:474, SEC22B:phospholipid is ~1:427 and SNAP23:phospholipid is ~1:215. The FRET assay showed that the lipid mixing between these adiposomes can be driven by full-length SNAREs (Fig. 4f, g). Besides, this lipid mixing was abolished in the absence of any of these SNAREs (Fig. 4f, g). Taken together, these results demonstrate that STX18, SNAP23 and SEC22B together directly drive the lipid mixing of adiposomes.

As the lipid mixing assay does not show the core TG exchange between adiposomes directly, we further performed the in vitro FRET-based content mixing assay of adiposomes (Fig. 4h). We reconstituted STX18–SNAP23-adiposomes encapsulated with Cy5-labeled DAG, and SEC22B-adiposomes encapsulated with Cy3-labeled DAG. The content mixing between adiposomes can be monitored by FRET built up between Cy5-DAG and Cy3-DAG. The SDS-PAGE showed successful reconstitution of these SNARE proteins on the adiposomes (Fig. 4i). The content mixing assay showed that in the presence of a full set of STX18, SEC22B and SNAP23, the adiposomes fused, but not in the reaction missing SNAP23 (Fig. 4j, k).

Altogether, our experiments including the lipid mixing assay and content mixing assay demonstrate that STX18–SNAP23–SEC22B drives membrane fusion of adiposomes.

### CIDEC/FSP27 promotes lipid mixing between adiposomes driven by STX18–SEC22B–SNAP23

**Fig. 5:**
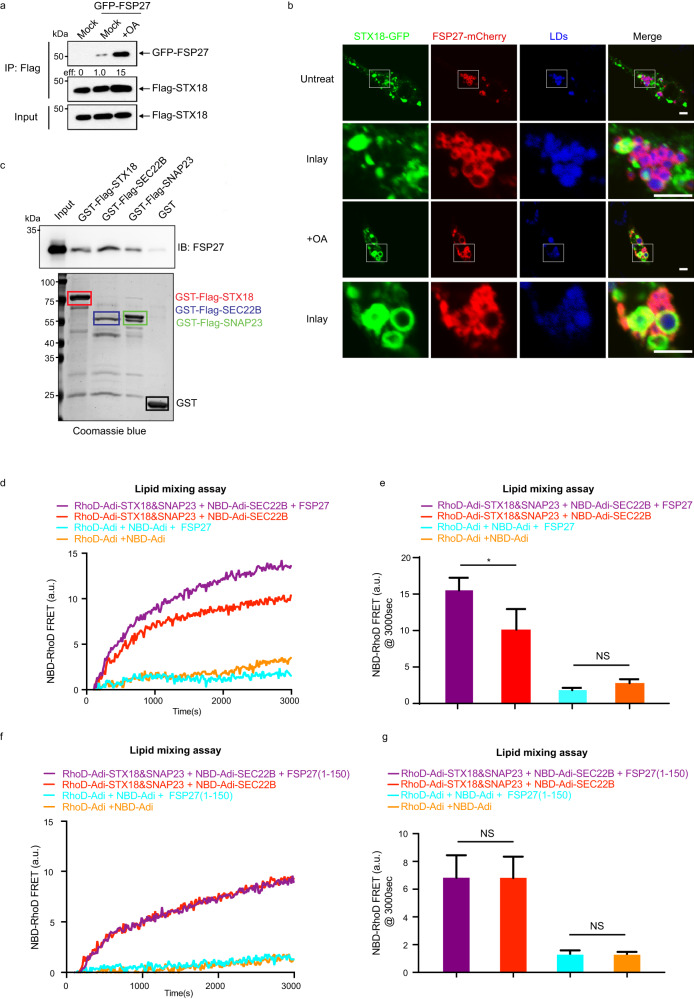
CIDEC/FSP27 promotes lipid mixing between adiposomes driven by STX18–SEC22B–SNAP23. **a** HEK293T cells were transfected with Flag-STX18 and GFP-FSP27 for 36 h, and then treated with or without 0.2 mM OA-BSA for 12 h. The cells were subjected to Flag IP followed by western blot analysis. **b** HepG2 cells were transfected with STX18-GFP and mCherry-FSP27 for 48 h, and then treated with 0.2 mM OA-BSA for 6 h. The LDs were stained by LipidTox Deep Red for 1 h after fixing the cells and the cells were analyzed via fluorescence. Scale bars, 10 μm (main images), 5 μm (inset). **c** Interaction between purified CIDEC/FSP27 and GST-Flag-tagged SNAREs using an in vitro GST pull-down assay followed by western blot. **d** Lipid mixing between adiposomes was measured from the development of FRET between NBD-labeled lipids and Rhod-labeled lipids. The NBD-adiposomes were reconstituted with SEC22B or not, while the RhoD-adiposomes were reconstituted with STX18, SNAP23 or not. The assays were performed in the presence of CIDEC/FSP27 or not. The reactions were recorded at 37 °C. **e** Quantification of lipid mixing experiments of **d**. Bars represent averages of the FRET signal observed after 3000 s in three independent experiments. Error bars represent SDs. Unpaired *t*-test; *P < 0.05, NS, not significant. **f** Lipid mixing between adiposomes was measured from the development of FRET between NBD-labeled lipids and Rhod-labeled lipids. The NBD-adiposomes were reconstituted with SEC22B or not, while the RhoD-adiposomes were reconstituted with STX18, SNAP23 or not. The assays were performed in the presence of CIDEC/FSP27 (1–150) or not. The reactions were recorded at 37 °C. **g** Quantification of lipid mixing experiments of **f**. Bars represent averages of the FRET signal observed after 3000 s in three independent experiments. Error bars represent SDs. Unpaired *t*-test; NS, not significant.

Previously, it was reported that CIDEC/FSP27 localized at LD contact sites and facilitated the LD fusion/coalescence. Its C-terminal sequence (a.a. 179–217) is responsible for LD targeting^8^, while its CIDE-N domain (a.a. 39–119) forms homodimers and defect of this homodimerization reduced CIDEC/FSP27’s activity in promoting the large LD formation^30^. We investigated the interplay between CIDEC/FSP27 and LD SNAREs in LD fusion. We first examined the interaction between CIDEC/FSP27 and SNAREs. Flag-STX18 interacted with GFP-FSP27 in HepG2 cells and this interaction was significantly enhanced upon OA treatment (Fig. 5a). Next, we tested whether CIDEC/FSP27 co-localized with STX18 on LDs in HepG2 cells. Without OA treatment, FSP27-mCherry localized on LDs, but STX18-GFP did not; thus we barely detected the co-localization between STX18 and CIDEC/FSP27. However, upon OA treatment, STX18-GFP was enriched on LDs, and co-localized well with FSP27-mCherry on LDs stained by LipidTox Deep Red (Fig. 5b). Moreover, we purified CIDEC/FSP27 to examine its direct binding with SNAREs. The pull-down experiments showed that the full-length CIDEC/FSP27 bound to GST-Flag-STX18, GST-Flag-SEC22B, and GST-Flag-SNAP23 (Fig. 5c). Furthermore, we purified CIDEC/FSP27 to assess its function in lipid mixing assay. CIDEC/FSP27 alone failed to drive lipid mixing of naked adiposomes, but CIDEC/FSP27 made lipid mixing of SNAREs-reconstituted adiposomes more efficient (Fig. 5d, e). By analyzing CIDEC/FSP27 purified from *E. coli*, we found that the purified CIDEC/FSP27 fraction is a mixture of full-length CIDEC/FSP27 and a truncated form which turns out to be its N-terminal fragment (1–150) by MS analysis (Supplementary Fig. S4a). To investigate the possibility that CIDEC/FSP27 (1–150) plays a role in lipid mixing of adiposomes, we cloned and purified the CIDEC/FSP27 (1–150) from *E. coli*. The results showed that no significant enhancement effect was observed when we used purified CIDEC/FSP27 (1–150) protein in the lipid mixing assay (Fig. 5f, g). We used DLS to examine the tethering function of CIDEC/FSP27 purified from *E. coli* on adiposomes, and interestingly, we found that adiposomes clustered without SNAREs, and this clustering effect was reversed with the addition of proteinase K (Supplementary Fig. S4b, c). Together, these results suggest that CIDEC/FSP27 could promote LD fusion driven by STX18–SNAP23–SEC22B by tethering adiposomes.

### STX18 mediates LD growth in mouse liver

**Fig. 6:**
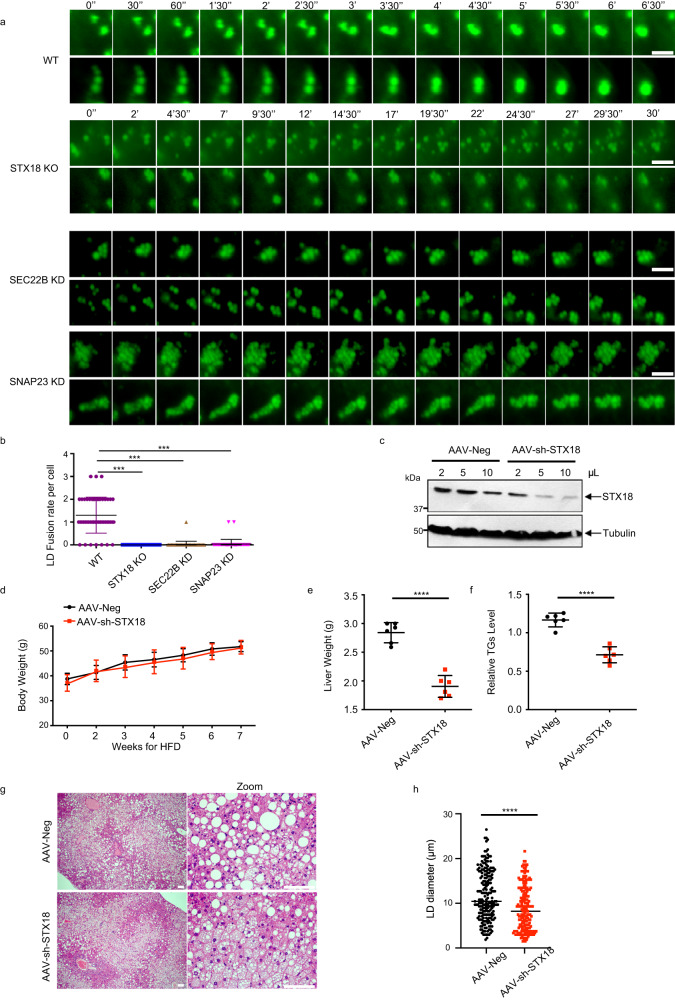
STX18 mediates LD growth in mouse liver. **a** HepG2 cells were treated with siNC, siSEC22B or siSNAP23. After 2 days, together with STX18 KO HepG2, they were treated with 0.2 mM OA-BSA for 2 h. LDs were stained by BODIPY 493/503 C_12_, and then cells were subjected to live-cell imaging. Scale bars, 5 μm. **b** Quantification of fusion rate per cell (*n* > 25 cells) from **a**. Student’s *t*-test; ***P < 0.001. **c** Male C57BL/6 mice (8 weeks of age) were injected with 2 μL, 5 μL or 10 μL of AAV-Neg or AAV-shSTX18 with vector titers at 1 × 10^10^ genomic copies/μL via tail veins for 4 weeks. Livers were excised and analyzed via western blot with STX18 antibody. **d** Male C57BL/6 mice (8 weeks of age) (*n* = 6) were injected with 1 × 10^11^ AAV-Neg or AAV-shSTX18 genomic copies via tail veins for 4 weeks and were fed with HFD for 7 weeks. Body weight was examined every week. **e** Liver weight from **d** was examined (*n* = 6). Unpaired *t*-test; ****P < 0.0001. **f** Total liver TG levels from **d** were examined (*n* = 6). Unpaired *t*-test; ****P < 0.0001. **g** Livers from **d** were excised, fixed and analyzed via histology (HE staining). Scale bars, 50 μm. **h** Statistical analysis of LD diameter in **g** (*n* > 200 LDs). Unpaired *t*-test; ****P < 0.0001.

STX18–SNAP23–SEC22B SNARE complex displays fusogenic activity in vitro, which prompted us to investigate their roles as a fusion machinery for LD coalescence in vivo. We first used live-cell imaging to document the fusion events in WT HepG2 cells, STX18 KO cells, SEC22B KD cells, and SNAP23 KD cells, respectively. Our results showed that compared to WT cells, fusion events were much less in cells depleted of STX18, SNAP23, or SEC22B upon OA treatment up to 12 h (Fig. 6a, b). Next, we investigated whether depletion of STX18 affects LD acumulation in mouse liver. Mice were injected with AAV carrying shRNA against STX18 or mock-treated for 4 weeks to knock down STX18 in the liver (Fig. 6c), and the mice were further fed with HFD for another 7 weeks. No significant difference was observed in the body weight between the control and STX18-knockdown group (Fig. 6d), but knockdown of STX18 reduced the liver weight (Fig. 6e) and TG level (Fig. 6f). Importantly, the knockdown of STX18 resulted in smaller LDs under HFD condition (Fig. 6g, h). Given that STX18 depletion in HepG2 cells has no effect on lipid synthesis (Fig. 1h), the decrease of TG in mouse liver is likely due to defects in LD fusion. Besides, previous reports have suggested that LDs with smaller sizes are more susceptible to lipophagic internalization^31^. Consequently, the depletion of STX18 in the mouse liver resulted in the reduction of LD size, liver weight and TG levels, possibly due to increased lysosomal uptake and degradation of these smaller LDs following STX18 depletion. All these results indicated a critical role of STX18 in LD fusion, and STX18 depletion antagonizes LD growth upon HFD feeding.

## Discussion

SNARE proteins are proven to be the core of fusion machinery in many membrane fusion events; however, the fusion machinery in LD coalescence was not fully demonstrated before. In the present study, we identified a new set of SNAREs, STX18–SNAP23–SEC22B, to be essential for LD fusion. This notion is supported by the following evidence: (1) STX18, SNAP23, and SEC22B were identified in unbiased RNAi screening or MS proteomic analysis for SNAREs required for LD size control; (2) Depletion of STX18, SNAP23 or SEC22B affects the LD growth in cells, but not lipid biogenesis, lipolysis, and fatty acid uptake; (3) STX18, SNAP23 and SEC22B localize on LDs and their interaction is enhanced upon OA treatment; (4) STX18, SNAP23 and SEC22B assemble into a SNARE complex in vitro; (5) STX18–SNAP23–SEC22B exhibits both lipid mixing and content mixing activity in reconstituted adiposome system; (6) STX18, SNAP23 and SEC22B bind with CIDEC/FSP27, a previously identified factor involved in LD size control, and their fusogenic activity is enhanced by CIDEC/FSP27; (7) Depletion of STX18 in mouse liver reduces LD size and TG level. For a long time, whether SNAREs can drive LD fusion has been under debate. In this work, we addressed this fundamental question by identifying STX18–SNAP23–SEC22B as the key machinery in LD fusion.

We observed a pivotal role of STX18–SNAP23–SEC22B in LD size control, LD targeting, and LD fusion in HepG2 cells, but we still cannot exclude the possibility that SNAREs other than this set are also involved in LD fusion. Besides, SNAREs might regulate different aspects of LD dynamics depending on the cell type. For instance, in STX18 KO 3T3-L1 preadipocytes, both small and medium/large LDs are reduced, implying that STX18, in conjunction with BNIP1 and USE1, might play a role in LD biogenesis in these cells^14^. Moreover, previous studies reported that depletion of Sec22b has moderate effect on LD morphology in 3T3-L1 cells^14^ and HeLa cells^32^. In our study in HepG2 cells, STX18 translocates to LDs where it complexes with SEC22B and SNAP23 to mediate LD fusion. Whether STX18–SNAP23–SEC22B mediates LD fusion in other tissues apart from the liver still needs further investigation.

STX18 was reported as a ER-resident t-SNARE involved in targeting and fusion of Golgi-derived retrograde transport vesicles with the ER^33^ and also as component of the tethering complex between ER and LDs^14^. We found that a significant portion of STX18 also localizes on LDs. Different from most organelle membrane, LD membrane is composed of single leaflet with the lipid hydrophobic tail embedded in the hydrophobic TG core of LD. How STX18 folds on LD membrane is important for its fusogenic activity. We figured out the topology of STX18 on in vitro reconstituted adiposomes. The cysteine accessibility assay suggests that STX18 spans the phospholipid monolayer twice, with its N-terminal soluble part and C-terminal hydrophilic residues facing cytosol. It is well known that the thickness of phospholipid bilayer is ~3–4 nm, and the thickness of phospholipid monolayer is therefore ~1.5–2 nm. There are 23 residues in TMD of STX18 (311–333) and two hydrophilic residues (334D and 335S) following the TMD. The I-Tasser program predicted that the TMD of STX18 can be two split short α-helices with a flexible linker in between. As each amino acid residue in an α-helix corresponds to a translation of 1.5 Å along the helical axis, these short α-helices with 10 amino acids predicted by I-Tasser, should be long enough to cross the phospholipid monolayer. The cysteine accessibility results (Fig. 3e) proved our hypothesis that TMD of STX18 spans the phospholipid monolayer twice, with both N-terminal and C-tail hydrophilic residues facing the cytosol. Besides, STX18 with C-terminal fused GFP is efficiently targeted to LD (Fig. 3a, b), which also suggests that the tail of STX18 is exposed to cytosol rather than facing the hydrophobic core of LD in cells.

**Fig. 7:**
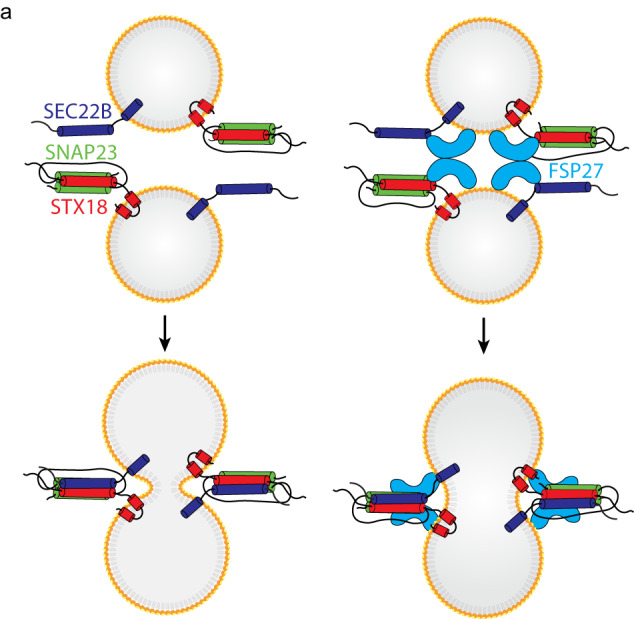
The hypothesis of LD fusion mediated by STX18, SNAP23, and SEC22B. **a** STX18 (Qa), SNAP23 (Qbc), and SEC22B (R) can form a SNARE complex by zipping their SNARE domains to drive the fusion between two adjacent LDs. The CIDEC/FSP27 can promote LD fusion by tethering two LDs.

Although SNARE proteins constitute the minimum fusion machinery, the tethering factors and the accessory proteins are also crucial to bridge two membrane compartments and boost fusion efficiency and precision. Previously, CIDEC/FSP27 was reported to locate at LD contact sites and promote LD growth. Its C-terminal sequence (a.a. 179–217) is responsible for LD targeting^8^, while its CIDE-N domain (a.a. 39–119) forms homodimers^30^. Therefore, CIDEC/FSP27 possibly serves as a membrane tether in LD fusion. In the present study, we showed that CIDEC/FSP27 interacted with LD SNAREs (Fig. 5a, c), and co-localized with STX18 upon OA treatment (Fig. 5b). Besides, CIDEC/FSP27 promoted LD lipid mixing driven by STX18–SNAP23–SEC22B (Fig. 5d, e). Moreover, CIDEC/FSP27 has no fusogenic activity by itself (Fig. 5d, e) but can promote adiposome clustering without SNAREs (Supplementary Fig. S4b, c). Furthermore, CIDEC/FSP27-promoted LD fusion could be abolished by depletion of STX18, SNAP23, or SEC22B in vivo (Fig. 1e, f). Therefore, we propose a model in which CIDEC/FSP27 dimer tethers LDs/adiposomes to close proximity and promotes STX18–SNAP23–SEC22B-mediated membrane fusion (Fig. 7a). It is worth noting that we did not exclude the possibility that CIDEC/FSP27 might have functions other than membrane tether in LD fusion. Li’s group recently proposed that the gel-like condensation of CIDEC/FSP27 generates lipid-permeable plates for LD fusion^11^. Whether CIDEC/FSP27 provides a hub to recruit SNAREs and other regulators to this plate and function together for fusion will be an attractive model to test.

In addition to the fusion machinery on the LD surface, the lipid compositions may also contribute to LD fusion. It is reported that PC inhibits fusion by lowering surface tension, and PA promotes fusion by increasing membrane curvature^6,34^. How STX18–SNAP23–SEC22B cooperates with lipid components on LDs will be further investigated. Furthermore, we showed that STX18 not only affects LD fusion on synthetic adiposomes, but also significantly influences LD size in cultured cells and mouse livers. Targeting this fusion machinery could be a promising strategy to counter against liver with extra lipid accumulation as well as many other diseases or pathological conditions with aberrant lipid metabolism. So far, we only included SNAREs and tethering proteins in the adiposome membrane fusion system. However, for classic membrane fusion theory, Rab GTPase, tethering proteins, SM proteins, NSF/αSNAP, lipid compositions, and membrane curvature all play a role in guaranteeing the specificity and efficiency of the membrane fusion. With this basic fusion machinery in place, we can start to dissect the complex and delicate regulatory mechanism of LD fusion.

## Materials and methods

### Cell cultures

U_2_OS, HEK293T, HeLa, and Huh7 cells were cultured in DMEM supplemented with 10% FBS; HepG2 cells were cultured in DMEM/F12 media supplemented with 10% FBS. All cells were cultured at 37 °C with 5% CO_2_. STX18 KO HepG2, STX18 KO U_2_OS, STX18 KO HeLa, HEK293T cells stably expressing ZZ-Flag-STX18 and HEK293T cells stably expressing ZZ-Flag-SEC22B were constructed in our lab.

### Plasmid construction

PCDNA4.0-STX18-FLAG, PCDNA4.0-STX18△TMD-FLAG, and PCDNA4.0-STX18-△SNARE-FLAG were cloned into mammalian expression vector PCDNA4.0-FLAG. PCDNA5.0/FRT/TO-ZZ-Flag-SEC22B was cloned into mammalian expression vector PCDNA5/FRT/TO. PTY-STX18-GFP, PTY-GFP-SNAP23, PTY-GFP-SEC22B, and PTY-GFP-FSP27 were cloned into mammalian expression vector PTY-GFP. PLV-STX18-GFP was cloned into mammalian expression vector PLV-GFP. PLV-FSP27-mCherry was cloned into mammalian expression vector PLV-mCherry. The mCherry-Plin2 was constructed previously. LiveDrop-GFP was a gift from Dr. Peng Li (Tsinghua University).

For protein purification in *E. coli* expression system, STX18, STX18-SNARE, STX18-SNARE-TMD, STX18-SNARE-TMD-Cys, CIDEC/FSP27, and CIDEC/FSP27 (1–150) were cloned into pGEX-4T-1. SEC22B and SNAP23 were cloned into pGEX vector. SEC22B-SNARE was cloned into pET28a vector. The SNARE domains of STX18 (243–305) and SEC22B (134–194) were cloned into pETDuet-1 vector. The SNARE domains of SNAP23 (14–76 and 146–208) were cloned into pACYCDuet-1 vector.

### Generation of STX18 KO cell line

The STX18 KO HepG2 cell line was generated by CRISPR/Cas9 genome editing. The targeting sequence of *STX18* is 5′-AAGACGCGGAACAAGGCGCT-3′. The sgRNAs were cloned into PX330 vectors and HepG2 cells were transfected by the resulting PX330 coding CAS9 and gRNA using Lipofectamine 3000 (Invitrogen). Later 48 h, cells were treated with 1 μg/mL puromycin for 3–5 days. Surviving cells were then isolated as single-cell clones by limiting dilution and the knockout clones were identified by western blot. STX18 KO U_2_OS cell line and STX18 KO HeLa cell line were generated in the same way.

### Generation of HEK293T cell line stably expressing ZZ-Flag-STX18 or ZZ-Flag-SEC22B

To generate HEK293T cell line stably expressing ZZ-Flag-STX18, packaging plasmids psPAX2, pMD2.G, and lentiviral plasmid lipodetector were transfected into 293T cells to package virus carrying ZZ-Flag-STX18 plasmids. 48 h after transfection, the medium containing mature virus was filtered by 0.45 µm filter, and then used to infect adherent HEK293T cells with the help of polybrene. A pool of HEK293T cells expressing ZZ-Flag-STX18 was used. HEK293T cells stably expressing ZZ-Flag-SEC22B was generated in the same way, except that the ZZ-Flag-SEC22B was used instead of ZZ-Flag-STX18.

### Antibodies

Anti-STX18 (sc-293067), anti-SNAP23 (sc-374215), anti-SEC22B (sc-101267), anti-STX5 (sc-365124), anti-VAMP4 (sc-365332), and anti-BAP31 (sc-393810) were obtained from Santa Cruz Biotechnology. Anti-Sec31A (A9321) was obtained from ABclonal. Anti-FSP27 was obtained from Peng Li. Anti-Flag (A8592) was obtained from Sigma-Aldrich. Anti-tubulin (E7-S) was obtained from Developmental Studies Hybridoma Bank. Anti-CHOP (2895) was obtained from Cell Signaling Technology. Anti-GFP (632381) was obtained from Clontech. Peroxidase AffiniPure Goat Anti-Mouse IgG (H + L) (115-035-003) and Peroxidase AffiniPure Goat Anti-Rabbit IgG (H + L) (111-035-003) were obtained from Jackson Immuno Research. Goat anti-Mouse IgG (H + L) Secondary Antibody, Alexa Fluor® 488 conjugate (A32723) were obtained from Thermo Fisher Scientific. Anti-Flag M2 Affinity Gel (A2220) was obtained from Sigma-Aldrich. Protein A/G PLUS Agarose (sc-2003) was obtained from Santa Cruz Biotechnology.

### Chemicals and commercial assays

Oleic Acid-Albumin from bovine serum (O3008), Tunicamycin (T7765), Thapsigargin (T9033), DOPC (850375 C), POPE (850757), NBD PE (810144), Liss RhoD PE (810158), Nile Red (72485), 3× FLAG Peptide lyophilized powder (F4799) was obtained from Sigma-Aldrich. Lipofectamine 3000 (L3000-015) was obtained from Thermo Fisher Scientific. BODIPY 558/568 C_12_ (D3835), BODIPY 493/503 (D3922), HSC LipidTox Deep Red neutral lipid stain (H3447) and TRlzol reagent (15596026) were obtained from Invitrogen. Protease inhibitor Cocktail tablets (04693132001) and (K1010) were obtained from Roche and APExBIO, respectively. iTaq™ Universal SYBR® Green Supermix (1725120) was obtained from Bio-Rad. BCA protein assay kit (23227) was obtained from Thermo Fisher Scientific. β-OG (O311) and FOS-CHOLINE-12 (F308) were obtained from Anatrace. DDM (DDM25) was obtained from GOLDBIO. Hoechst 33342 (40731ES10) was purchased from Yeasen. DAPI (P36962) was purchased from Thermo Fisher Scientific.

### Oligonucleotides

SMARTpool: siRNAs targeting ON-TARGETplus *STX18*, *SNAP23*, *SEC22B*, *ATG7*, and all other SNARE genes were purchased from Dharmacon.

Crisper *STX18* sgRNA to generate STX18 KO cell line is:

AAGACGCGGAACAAGGCGCT.

The mouse *STX18* shRNA sequence for AAV preparation is:

TTCTTGTCCACCACGCGCT.

### Co-IP and western blot

Cells were harvested and lysed with TAP buffer (20 mM Tris-HCl, pH 7.5, 150 mM NaCl, 0.5% NP-40, 1 mM NaF, 1 mM Na_3_VO_4_, 1 mM EDTA, protease inhibitor cocktail) for 30 min on ice. The supernatants were collected by centrifugation at 1,3000 rpm for 10 min at 4 °C and precleared by incubating with protein A/G plus agarose for 1 h at 4 °C. For Flag IP, beads were added into supernatants and incubated overnight. Beads were washed three times with TAP buffer and boiled at 100 °C for 10 min in SDS protein loading buffer and analyzed by western blot. Protein concentration was determined based on the Bradford method using the Bio-Rad protein assay kit. Equal amounts of protein were separated by SDS-PAGE and electrophoretically transferred onto a nitrocellulose membrane. After blocking with 5% nonfat milk in PBST, the membrane was incubated with the primary antibody, followed by HRP-conjugated goat anti-rabbit or anti-mouse IgG.

### RT-qPCR

Total RNA was isolated from cells and frozen tissues by homogenization in Trizol. Reverse transcription was performed using M-MLV reverse transcription reagents (Promega). cDNA was subjected to qPCR analysis.

The primers are:

*DGAT1* forward, TATTGCGGCCAATGTCTTTGC,

*DGAT1* reverse, CACTGGAGTGATAGACTCAACCA;

*DGAT2* forward, ATTGCTGGCTCATCGCTGT,

*DGAT2* reverse, GGGAAAGTAGTCTCGAAAGTAGC;

*FASN* forward, AAGGACCTGTCTAGGTTTGATGC,

*FASN* reverse, TGGCTTCATAGGTGACTTCCA;

*xbp1s* forward, TGCTGAGTCCGCAGCAGGTG,

*xbp1s* reverse, GCTGGCAGGCTCTGGGGAAG;

*BNIP1* forward, CTGGAGCAGTTGGCTAAAGAGC,

*BNIP1* reverse, GCAGGTGAGATTAGCTTTCCTCC;

*USE1* forward, AGCGACATCAGAACCTCCAGGA,

*USE1* reverse, TCCAGGTTCTGGTCCGCCATTT;

*GAPDH* forward, GAAGGTGAAGGTCGGAGT,

*GAPDH* reverse, GAAGATGGTGATGGGATTTC.

### Immunofluorescence analysis

Cells were rinsed in PBS, fixed with 4% paraformaldehyde (PFA) for 15 min, permeabilized with 0.1% saponin in PBS for 10 min, blocked with 10% FBS in PBS for 30 min, followed by incubation with primary antibody for 2 h, washed three times with PBS, and incubated with fluorescently labeled secondary antibody for 1 h. Cells were then washed with PBS three times. ProLong^TM^ Diamond Antifade Mountant with DAPI (Thermo Fisher Scientific, P36962) was used to stain the nucleus. Images were acquired on a confocal laser microscope (Zeiss LSM880 Airyscan) using a 60× oil-immersion objective lens. Images were processed with ImageJ software. The microscopy data are quantified in a blinded manner.

### LD staining

Cells were treated with 0.2 mM OA-BSA for the indicated time, and then LDs were stained by BODIPY 493/503 or BODIPY 558/568 C_12_ for 1 h at 37 °C with 5% CO_2_ before being fixed with 4% PFA. Cells were also stained by Nile red or lipidTox Deep red for 0.5 h if required.

### Ultrathin section

The fresh adiposomes were quickly mixed with gelatin and solidified on ice. The solidified samples were cut into blocks of ~1 mm^3^. The blocks of adiposomes were fixed with 2.5% glutaraldehyde at room temperature (RT) for 1 h. After the blocks were washed three times with PB buffer, they were fixed with 1% osmic tetroxide at RT for 2 h. After washing three times with PB buffer, the fixed blocks were dehydrated with ethanol and acetone, and then embedded in resin. The blocks were polymerized at 37 °C for 8 h, then at 65 °C for 48 h, and subjected to untrathin slicing at 70 nm. Finally, ultrathin sections were stained with uranyl acetate for 7 min. Micrographs were recorded on a transmission electron microscope (Thermo Fisher/FEI Talos L 120 C).

### Immunoelectron microscopy

The HepG2 cells transfected with plv-STX18-GFP were cultured on 3 mm sapphire discs. A Sapphire disc was placed with cells facing up on a flat aluminum planchette and another aluminum planchette with 25-µm depth inner space was used as a cover. The spaces between the two aluminum planchettes were filled with 1-hexadecane. Then the samples were frozen immediately using the EM ICE high-pressure freezing machine (Leica) and rapidly transferred into liquid nitrogen for storage. After all of the samples were frozen, the samples were transferred into the EM ASF2 (Leica) for substitution. Samples were incubated for 48 h in acetone containing 0.2% UA at –90 °C. Then the temperature was raised to –50 °C for 4 h. After incubation in acetone containing 0.2% UA for another 12 h, the temperature was raised to –30 °C for 4 h. After 2 h incubation at –30 °C, the samples were rinsed three times with pure acetone (15 min each). Then the samples were gradually infiltrated in HM20 resin with grades of 25%, 50%, 75% and pure resin (1 h each) at –30 °C. After being infiltrated in pure resin overnight, the samples were embedded in gelatin capsules. The samples were polymerized under UV light for 48 h at –30 °C and 12 h at 25 °C. After polymerization, the samples were trimmed and ultrathin sectioned with a microtome (Leica UC7). Serial thin sections (100 nm thick) were collected onto formvar-coated nickel grids. The formvar-coated nickel grids with sections were incubated in 0.01 M PBS containing 1% BSA, 0.05% Triton X-100, and 0.05% Tween 20 for 5 min. Then the sections were incubated with the primary antibody (rabbit anti-GFP) diluted in 0.01 M PBS containing 1% BSA and 0.05% Tween 20 at 4 °C overnight. After being washed 6 times (2 min each) with 0.01 M PBS, the sections were incubated with the secondary antibody (goat anti-rabbit conjugated with 10 nm gold) diluted in 0.01 M PBS containing 1% BSA and 0.05% Tween 20 (1:50) for 2 h at RT. After being washed 6 times (2 min each) with 0.01 M PBS and 4 times (2 min each) with distilled water, the section was dried at RT and examined under a transmission electron microscope (Thermo Fisher/FEI Talos L 120 C).

### Lipolysis (3T3-L1) colorimetric assay kit

In 6-cm culture dish, the HepG2 cells were treated with siNC or siSTX18 for 45 h. Then cells were washed three times with PBS and incubated in phenol red-free, serum-free F12 medium (Gibco) containing 1% fatty acid-free BSA in the presence or absence of 500 nM isoproterenol (Sigma-Aldrich, 1351005) for 3 h. The amount of glycerol released into culture medium was measured by commercial kit, Free Glycerol Reagent (Sigma-Aldrich, F6428).

### Fatty acid uptake assay

The HepG2 cells were treated with siNC or siSTX18 for 48 h. At least 10,000 cells per well were cultured in 96-well plate. Fatty acid uptake level was analyzed via Fatty Acid Uptake Assay Kit (MAK156) from Sigma.

### Measurement of LD fusion rates in HepG2 cells

HepG2 cells were grown on glass-bottom confocal dishes, and were transfected with siNC, siSEC22B, or siSNAP23 for 48 h. Cells were further treated with 0.2 mM OA-BSA for 2 h and stained with BODIPY 493/503 for another 30 min. Cells were imaged and captured by Olympus FV3000 Confocal Microscope with cell culture system. The dishes were monitored and addressed by the Z drift compensator system. Images were acquired using a 60×/1.4 oil objective with Z series and time-series. Time lapses was performed with 30-s interval for 120 cycles. Cells were imaged by using the 488 nm for BODIPY 493/503. Acquired images were processed with software FV31S-DT to analyze the fusion events. The LD fusion events were quantified from 25 cells of three biological replicates.

### Animal experiments

Male C57BL/6 mice (8 weeks of age) were housed under a 12 h light/dark cycle with access to standard rodent chow diet (2916, Teklad) and water. AAVs (serotype 8, transduce liver) were custom packaged at SignaGen Laboratories. The mouse *STX18* shRNA sequence for AAV preparation is TTCTTGTCCACCACGCGCT. STX18-shRNA or GFP-shRNA AAV was delivered by tail vein injection with vector titers at 1 × 10^10^ genomic copies/μL. Mice injected with 10 μL AAV vectors were studied at least 4 weeks after AAV injection to achieve maximal knockdown efficiency of STX18. Mice were further fed with HFD (60% kcal from fat, Bio-Serv) for 7 weeks. All animal studies and experimental procedures were approved by the Animal Care and Use Committee of the animal facility at UT Southwestern Medical Center.

### Immunohistochemistry

Livers were excised and fixed overnight in 10% PBS-buffered formalin. Then tissues were rinsed with 50% ethanol three times, embedded in paraffin blocks by the University of Texas Southwestern Medical Center Molecular Pathology Core, and sliced 5 μm each section. The slices were rinsed 3 times for 5 min each in PBS, pH 7.4, and trichrome staining was processed by University of Texas Southwestern Medical Center Molecular Pathology Core via standard procedure.

### Cloning, expression, and purification of full-length STX18, SEC22B, and SNAP23

Full-length STX18 was cloned into pGEX-4T-1 vector between *Bam*HI and *Not*I, with a GST-TEV-3×FLAG tag at its N-terminal sequence. The construct was transformed into *E. coli* BL21 (C43) cells. The target protein expression was induced by 0.1 mM IPTG at 16 °C for 16 h. The harvested cells were lysed at lysis buffer (20 mM HEPES, pH 7.4, 500 mM NaCl, 1 mM EDTA) supplemented with Protease Inhibitor Cocktail (APExBIO) and centrifuged at 8000 rpm in JA 14 rotor (Beckman Coulter) for 25 min to remove inclusion bodies and cell debris. The supernatant was collected and re-centrifuged at 40,000 rpm in Ti45 rotor (Beckman Coulter) for 2 h. The pellet containing the membrane was homogenized by Dounce homogenizer (KONTES GLASS) in lysis buffer. The membrane proteins were extracted by solubilizing the membranes with 2% Dodecylmaltoside (Anatrace) and then the samples were centrifuged at 40,000 rpm in Ti45 rotor for 60 min. The supernatant was incubated with Glutathione Sepharose 4B (GE Healthcare) overnight, and then the resins were washed by wash buffer (20 mM HEPES, pH 7.4, 500 mM NaCl, 1 mM EDTA, 0.1% Fos-Choline-12). The protein was treated with TEV protease on the beads at 4 °C for 2 h, and then eluted by elution buffer (20 mM HEPES, pH 7.4, 300 mM NaCl, 1 mM EDTA, 0.1% Fos-Choline-12). The full-length SEC22B was cloned into pGEX vector between *Eco*RI and *Xho*I with a GST tag at its N-terminus and a TEV cleavage site following GST tag. The expression and purification of SEC22B are the same as that of full-length STX18. The full-length SNAP23 was cloned into pGEX vector between *Eco*RI and *Xho*I with a GST tag at its N-terminus and a TEV cleavage site following GST tag. The construct was transformed into *E. coli* BL21 (DE3) cells. Overexpression of the protein was induced by the addition of 0.1 mM IPTG at 16 °C for 16 h. The harvested cells were lysed by high pressure homogenizer in lysis buffer (20 mM HEPES, pH 7.4, 500 mM NaCl, 1 mM EDTA) supplemented with Protease Inhibitor Cocktail (APExBIO) and centrifuged at 40,000 rpm in Ti45 rotor for 60 min. The cleared supernatant was incubated with Glutathione Sepharose 4B (GE Healthcare) at 4 °C overnight. The resins were washed with lysis buffer. The target protein was treated with TEV protease on the beads at 4 °C for 2 h, and then eluted by elution buffer (20 mM HEPES, pH 7.4, 300 mM NaCl, 1 mM EDTA). The protein was concentrated to the desired concentration if needed.

### Cloning, expression, and purification of STX18-SNARE and SEC22B-SNARE

STX18-SNARE (243–305) was cloned into pGEX-4T-1 vector between *Bam*HI and *Not*I, with GST-TEV-FLAG tag at its N-terminal sequence. The construct was transformed into *E. coli* BL21 (DE3) cells. Overexpression of the protein was induced by addition of 0.1 mM IPTG at 16 °C for 16 h. The harvested cells were lysed with lysis buffer (20 mM HEPES, pH 7.4, 500 mM NaCl, 1 mM EDTA) supplemented with Protease Inhibitor Cocktail (APExBIO) and centrifuged at 40,000 rpm in Ti45 rotor for 60 min. The cleared supernatant was incubated with Glutathione Sepharose 4B (GE Healthcare) at 4 °C overnight. The beads were washed with lysis buffer. The protein was treated with TEV protease on the GST agarose at 4 °C for 2 h, and then eluted by lysis buffer. Gel filtration chromatography (Superdex 75 increase 10/300 GL, GE Healthcare) was applied to further purify the protein in S75 buffer (20 mM HEPES, pH 7.4, 150 mM NaCl, 1 mM EDTA). The protein fractions were collected and concentrated to the desired concentration. SEC22B-SNARE (134–194) was cloned into pET28 vector between *Nde*I and *Eco*RI, with 6×His tag at its N-terminal sequence. The construct was transformed into *E. coli* BL21 (DE3) cells. Overexpression of the protein was induced by the addition of 0.1 mM IPTG at 16 °C. The cells were harvested after induction for 16 h, and resuspended in lysis buffer (20 mM Tris-HCl, pH 8.0, 500 mM NaCl, 10 mM Imidazole) supplemented with Protease Inhibitor Cocktail (APExBIO). The cells were lysed by high pressure homogenizer (ATS Engineer Inc., China) and centrifuged at 40,000 rpm for 60 min. The pellet was resuspended in urea buffer (20 mM Tris-HCl, pH 8.0, 500 mM NaCl, 6 M urea, 1 mM β-mercaptoethanol, 20 mM Imidazole) at RT for 2 h and centrifuged again at 20,000 rpm for 45 min. The cleared supernatant was applied to Ni-NTA agarose beads (Qiagen). The resins were washed by urea buffer. The target protein was refolded on Ni-NTA agarose beads by gradually reducing the urea concentration to 0 M. Finally, the protein was eluted from Ni-NTA agarose beads by elution buffer (20 mM Tris-HCl, pH 8.0, 500 mM NaCl, 500 mM Imidazole) and concentrated to the desired concentration.

### Cloning, expression, and purification of STX18-SNARE-TMD, STX18-SNARE-TMD-Cys and SEC22B-SNARE-TMD

The cloning, expression, and purification of STX18-SNARE-TMD, STX18-SNARE-TMD-Cys, and SEC22B-SNARE-TMD are similar to those of full-length STX18, except that 1% β-OG was used instead of 0.1% Fos-Choline-12 in both wash buffer and elution buffer.

### Cloning, expression and purification of the SNARE complex

The SNARE domains of STX18 (243–305) and SEC22B (134–194) were cloned into pETDuet-1 vector, with SEC22B (134–194) containing N-terminal His tag inserted between *Bam*HI and *Not*I restriction sites, and STX18 (243–305) inserted between *Nde*I and *Xho*I restriction sites, respectively. The SNARE domains of SNAP23 (14–76 and 146–208) were cloned into pACYCDuet-1 vector, with SNAP23 (14–76) containing a N-terminal His tag inserted between *Bam*HI and *Sal*I restriction sites, and the SNAP23 (146–208) fragment inserted between *Nde*I and *Xho*I restriction sites, respectively. The two plasmids were co-transformed into *E. coli* BL21 (DE3) cells under the selection of both Ampicillin and Chloramphenicol. Expression of target proteins was induced by 0.1 mM IPTG at 16 °C for 16 h. The cells were harvested and resuspended in lysis buffer (20 mM HEPES, pH 7.4, 300 mM NaCl, 20 mM Imidazole) supplemented with Protease Inhibitor Cocktail (APExBIO). Then the cells were lysed and centrifuged at 40,000 rpm for 60 min. The cleared supernatant after centrifugation was loaded onto Ni-NTA agarose beads (Qiagen). The resin was washed with wash buffer (20 mM HEPES, pH 7.4, 300 mM NaCl, 50 mM Imidazole), and the target proteins were eluted with elution buffer (20 mM HEPES, pH 7.4, 300 mM NaCl, 500 mM Imidazole). Fractions containing the SNARE complex were pooled and concentrated using an Amicon Ultra-15 centrifugal filter with 10-kDa molecular mass cut off (Millipore) to reduce the volume. The complex was further purified by gel filtration chromatography (Superdex increase 75 10/300 GL, GE Healthcare) with SEC buffer (50 mM HEPES, pH 7.4, 150 mM NaCl, 0.5 mM TCEP). Fractions were analyzed by SDS-PAGE and MS.

### Cloning, expression and purification of CIDEC/FSP27 and CIDEC/FSP27 (1–150)

Full-length CIDEC/FSP27 was cloned into pGEX-4T-1 vector between *Bam*HI and *Not*I, with a GST-TEV-3×FLAG tag at its N-terminal sequence. The construct was transformed into *E. coli* BL21 (C43) cells. The target protein expression was induced by 0.1 mM IPTG at 16 °C for 16 h. The harvested cells were lysed by high pressure homogenizer (ATS) at lysis buffer (20 mM HEPES, pH 7.4, 500 mM NaCl, 1 mM EDTA, 1% Triton) supplemented with Protease Inhibitor Cocktail (APExBIO) and centrifuged at 40,000 rpm in Ti45 rotor (Beckman Coulter) for 60 min to remove cell debris. The supernatant was applied to Glutathione Sepharose 4B (GE Healthcare). The resin was washed by wash buffer (20 mM HEPES, pH 7.4, 500 mM NaCl, 1 mM EDTA, 1% β-OG). The target protein was treated with TEV protease on the beads at 4 °C for 2 h, and then eluted by elution buffer (20 mM HEPES, pH 7.4, 300 mM NaCl, 1 mM EDTA, 1% β-OG). The cloning, expression and purification of CIDEC/FSP27-N (1–150) are similar to those of the full-length CIDEC/FSP27, except that the 0.1% Fos-Choline-12 was used instead of 1% β-OG in both wash buffer and elution buffer.

### In vitro pull-down assays

The interaction between STX18, SEC22B, and SNAP23 was investigated using an in vitro GST pull-down assay. Initially, GST agarose beads were equilibrated with Wash Buffer (20 mM HEPES, pH 7.4, 300 mM NaCl, 1 mM EDTA, 1% OG, 0.1% FOS-Choline-12) and separately incubated with GST-Flag-tagged proteins: GST-Flag-STX18 (2.57 μM, 80 μL), GST-Flag-SNAP23 (9.8 μM, 45 μL), or GST protein control (98 μM, 5 μL) at 4 °C for 1 h. Following this, the GST agarose beads were washed with Wash Buffer and incubated separately with the indicated SNAREs: SEC22B (54.7 μM, 20 μL) for Fig. 2e, g, and SNAP23 (280 μM, 5 μL) for Fig. 2f at 4 °C for 1 h. Subsequently, the beads were washed again with Wash Buffer and subjected to protein denaturation. The interactions were analyzed using western blotting and Coomassie blue staining.

To detect the interaction between purified CIDEC/FSP27 and GST-Flag-tagged SNAREs, GST agarose beads were equilibrated with Wash Buffer (20 mM HEPES, pH 7.4, 300 mM NaCl, 1 mM EDTA, 1% OG) and incubated separately with GST-Flag-STX18 (2.57 μM, 100 μL), GST-Flag-SEC22B (7.8 μM, 30 μL), GST-Flag-SNAP23 (6.6 μM, 35 μL) and GST (98 μM, 10 μL) at 4 °C for 1 h. After being washed with Wash Buffer, the GST agarose beads were incubated separately with FSP27 (3.3 mg/mL, 5 μL) at 4 °C for 1 h. The GST agarose beads were then subjected to protein denaturation after being washed with Wash Buffer. The interactions were analyzed using western blotting and Coomassie blue staining.

### Naked adiposome preparation

Adiposomes were prepared following the previously published method with some modifications^28^. Two milligrams of total phospholipids (66.6% DOPC, 33.3% POPE) in chloroform was added to a 1.5 mL microcentrifuge tube, and the solvent was dried by nitrogen gas. 100 μL RB buffer (20 mM HEPES, pH 7.4, 100 mM KCl, 2 mM MgCl_2_) was added into the tube and 20 μL of TG extracted from mouse fat tissue was added on the top of the RB buffer before vortex. The tube containing lipids and buffer was vortexed for 24 cycles of 10 s on and 10 s off. Then, the milky lipid mixture was centrifuged at 20,000× *g* at 4 °C for 10 min. The fraction containing adiposomes formed a floating white band at the top of the tube. The underlying solution and pellet were removed. A total of 100 μL RB buffer was added to the remaining fraction containing adiposomes which were resuspended by vortex. The sample was centrifuged again at 20,000× *g* for 5 min, and the solution underneath and pellet, if present, were removed. These procedures were repeated for three times, and then the white band containing adiposomes was resuspended in 100 μL RB buffer. After centrifugation at 1000× *g* for 5 min, the adiposomes as milky solution underneath a floating white band were collected for further application. The phospholipid concentration can be measured according to the method described before^35^.

### Reconstitution of adiposomes for ensemble lipid mixing assay

Donor adiposomes with full-length STX18 or STX18-SNARE-TMD and SNAP23 contained 66.6% DOPC, 23.3% POPE and 10% NBD PE. Acceptor adiposomes with full-length SEC22B or SEC22B-SNARE-TMD contained 66.6% DOPC, 31.3% POPE and 2% Liss Rhod PE. Adiposomes with corresponding lipid compositions were made freshly as described above. Purified SNARE proteins were added to adiposomes to make STX18:SNAP23:Lipid = 1:1.9:83 for donor adiposomes, and SEC22B:Lipid = 1:36 for acceptor adiposomes. The concentration of β-OG was kept ~0.8%, and the mixtures were incubated at 4 °C for 60 min. Later, the mixtures were diluted to make concentration of β-OG around 0.4% and then dialyzed against the reaction buffer (25 mM HEPES, pH 7.4, 150 mM KCl, 0.5 mM TCEP) with 1 g/L Biobeads SM2 (Bio-Rad) overnight. The samples were centrifuged at 20,000× *g* for 5 min, and then the adiposomes floating on the top were taken for lipid mixing assays. To make control adiposomes, the SNAREs were replaced with reaction buffer.

### Lipid mixing assay with adiposomes

For lipid mixing assays, donor adiposomes (0.15 mM phospholipids) were mixed with acceptor adiposomes (0.15 mM phospholipids) with the addition of SNAP23 (20 μM) or CIDEC/FSP27 if required in a total volume of 200 μL. The FRET signal was measured by exciting NBD-PE fluorescence probe at 460 nm and observing the emission signal from Rhod-PE at 580 nm with a Spectrofluorometer (Varian Eclipse). All experiments were performed at 37 °C. All the experiments were repeated with three independent preparations. For quantification, we calculated the average fluorescence at 3000 s and the corresponding standard deviation (SD).

### Reconstitution of adiposomes for ensemble content mixing assay

We used in-house synthesized Cy3-DAG and Cy5-DAG as the content dye for adiposomes in FRET-based content mixing assay (see Supplementary information for synthesis procedures of Cy3-DAG and Cy5-DAG). The synthesized Cy3-DAG and Cy5-DAG were dissolved in TG to make 1% (m:v) working stock separately.

Adiposomes encapsulated with Cy3-DAG or Cy5-DAG were made similarly to adiposomes with TG, except that half of the TG volume was replaced by Cy3-DAG or Cy5-DAG working stock. Theoretically, the reconstituted Cy3-labeled adiposomes and Cy5-labeled adiposomes should end up with surrounding phospholipid layer at 66.6% DOPC, 33.3% POPE and encapsulated content at 0.5% Cy3-DAG or Cy5-DAG.

Purified SNARE proteins were added to freshly made Cy3-labeled adiposomes or Cy5-labeled adiposomes to make STX18:SNAP23:phospholipid = 1:1.9:83 for Cy3-labeled adiposomes, and SEC22B:phospholipid = 1:36 for Cy5-labeled adiposomes. The mixtures were kept in the buffer containing 25 mM HEPES, pH 7.4, 150 mM KCl, 0.5 mM TCEP, 0.8% β-OG, and incubated at 4 °C for 60 min. Later, the mixtures were diluted to make concentration of β-OG around 0.4% and then dialyzed against the reaction buffer (25 mM HEPES, pH 7.4, 150 mM KCl, 0.5 mM TCEP) with 1 g/L Biobeads SM2 (Bio-Rad) overnight. The samples were centrifuged at 20,000× *g* for 5 min, then the adiposomes floating on the top were taken for content mixing assays. For control adiposomes, the SNAP23 was replaced with reaction buffer.

### Content mixing assay with adiposomes

For content mixing assay, Cy5-labeled STX18–SNAP23-adiposomes (0.15 mM phospholipids) were mixed with Cy3-labeled SEC22B-adiposomes (0.15 mM phospholipids) with addition of SNAP23 (20 μM) in a total volume of 200 μL. For control group, Cy5-labeled STX18-adiposomes (0.15 mM phospholipids) were mixed with Cy3-labeled SEC22B-adiposomes (0.15 mM phospholipids) without addition of SNAP23 in a total volume of 200 μL. The FRET signal was measured by exciting Cy3 at 553 nm and observing the emission signal from Cy5 at 670 nm with a Spectrofluorometer (Varian Eclipse). All experiments were performed at 37 °C. All the experiments were repeated in three independent experiments. For quantification, we calculated the average fluorescence at 7200 s and the corresponding SD.

### Measurement of SNAREs:lipids ratio

We used the Stewart Method to measure the phospholipid concentration of adiposomes. To avoid fluorescence dye to interrupt the 472 nm absorbance reading, we made adiposomes as how we made the adiposomes in FRET assay except that there are no fluorescence dye included. On the other side, we made a standard curve by liposomes with the same phospholipid compositions (66.6% DOPC, 33.3% POPE), to get a function of absorbance reading at 472 nm in terms of phospholipid amount. Based on this function and the 472 nm reading of the adiposomes we freshly made, we can get its phospholipid amount. Furthermore, the protein amounts of the adiposome samples were estimated by comparing the corresponding SNARE protein bands with BSA bands on SDS-PAGE. Then the ratio between phospholipids and proteins can be calculated.

### Cryo-EM of adiposomes

For cryo-EM, 2 μL of fresh adiposome was applied to grids and blotted for 3 s in 100% humidity using a FEI Vitrobot Mark IV and then vitrified by quickly plunging into liquid ethane precooled with liquid nitrogen. Micrographs were recorded using a ceta 4 K × 4 K CCD in an Talos Arctica cryo-electron microscope operating at 200 kV.

### Cysteine accessibility assay with adiposome-associated STX18

The cysteine labeling assay was performed following the previously published method with some modifications^36^. Purified STX18-SNARE-TMD (243–335) or STX18-SNARE-TMD-Cys was reconstituted on freshly made adiposomes (66.6% DOPC, 33.3% POPE). The STX18-decorated adiposomes were isolated from free proteins by a co-floatation assay on a Histodenze gradients (40%:35%:30%). The top fractions containing STX18-decorated adiposomes were collected and analyzed by SDS-PAGE and Coomassie blue staining. Then these adiposomes were labeled with excess of Alexa Fluor 488 C5 maleimide dye (Thermo Fisher Scientific) in the absence or presence of 2% Triton X-100 at 4 °C overnight. The samples were separated by SDS-PAGE and analyzed by fluorescent imaging and Coomassie blue staining.

### Measurement of the secondary structure of protein by circular dichroism

Both the purified STX18 (243–335) and STX18 (243–335)-Cys were dissolved in stock buffer (20 mM HEPES, pH 7.4, 300 mM NaCl, 1 mM EDTA, 1% β-OG). The protein samples were diluted with ddH_2_O 15 times, and then diluted samples were applied to circular dichroism, using the diluted stock buffer as the baseline.

### Quantification and statistical analysis

Statistical parameters including the definition and exact values of *n*, distribution and deviation are reported in the figure legends. Data are expressed as means ± SD. The significance of the variability between different groups was determined by two-way analyses of variance using GraphPad Prism software. Error bars, SDs of two or three independent experiments. Student’s *t*-test, a *P* value of < 0.05 was considered statistically significant and a *P* value of > 0.05 was considered statistically non-significant (NS).

### Supplementary information

Supplementary information
