# Supplementary material for: Qa-SNARE syntaxin 18 mediates lipid droplet fusion with SNAP23 and SEC22B
Source: Cell Discov. 2023 Nov 21;9:115. doi: 10.1038/s41421-023-00613-4 (PMC10663520; doi:10.1038/s41421-023-00613-4)
Supplement: Supplementary file 1 — Supplementary information [file 41421_2023_613_MOESM1_ESM.pdf]

Supplementary Fig. S1

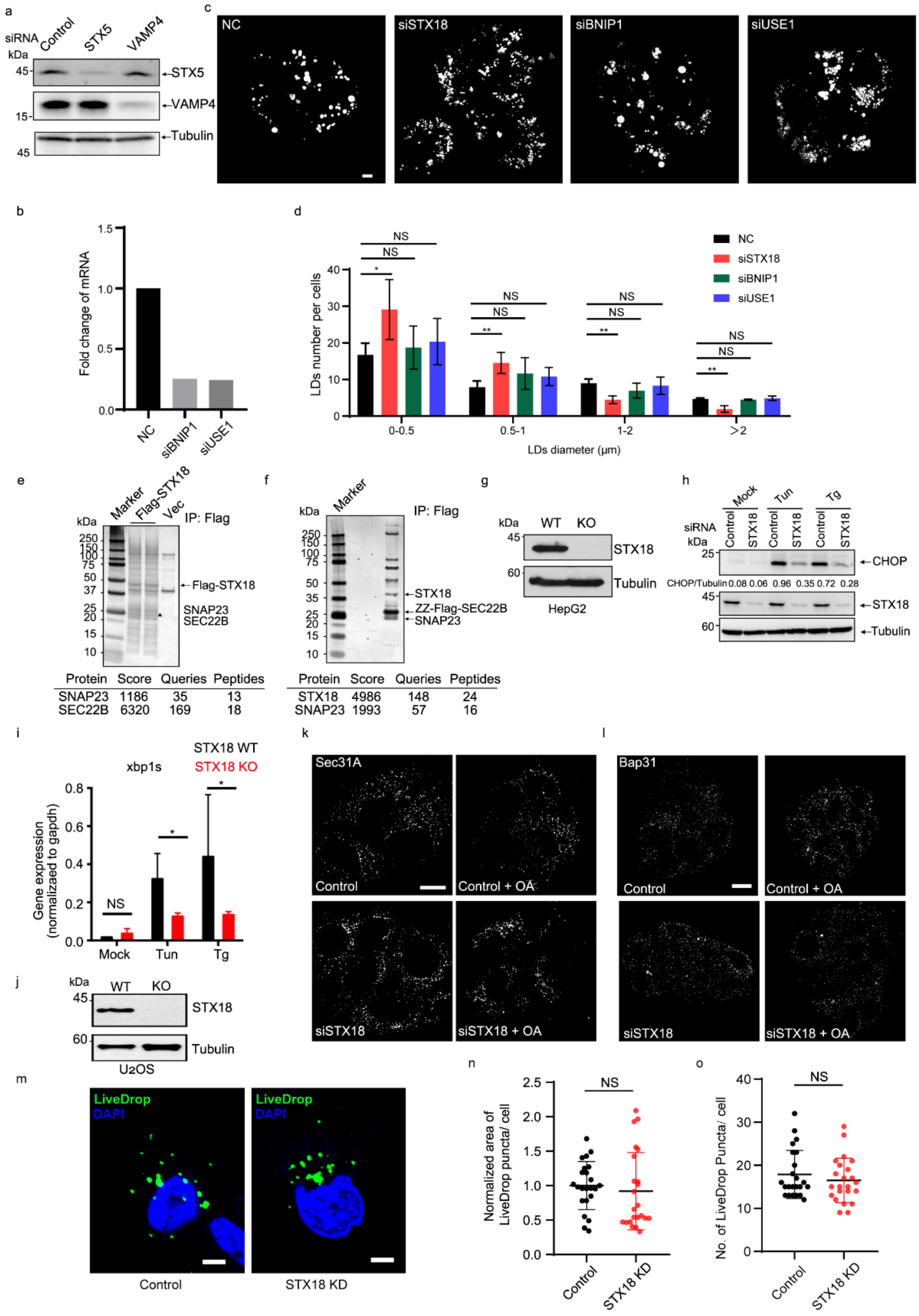

Supplementary Fig. S1: Identification of SNAREs STX18, SNAP23, SEC22B for LD size control.

a HepG2 cells were transfected with control, STX5, or VAMP4 siRNAs for 48 h and then cells were analyzed via Western blot.

b HepG2 cells were transfected with non-targeting (NC), BNIP1, or USE1 siRNAs for 48 h and mRNA levels of BNIP1 and USE was determined by qPCR.

c HepG2 cells were transfected with non-targeting, STX18, BNIP1, or USE1 siRNAs for 48 h and then treated with 0.2 mM OA-BSA for 12 h. LDs were stained by BODIPY-493/503 C_{12} for additional 1 h before fixing the cells. Cells were analyzed via fluorescence. Scale bar: 5 μm.

d Quantification of big LDs (≥2 μm) in Fig. S1c by analyzing average LD number in 50 cells. Unpaired t test; * $P < 0.05$, ** $P < 0.01$; NS, not significant.

e Silver staining of affinity-purified Flag-STX18 complex or vector alone in HEK293T cells stably expressing Flag-STX18. All the marked bands were identified by mass spectrometry. The asterisk showed the band position where SEC22B and SNAP23 were identified. The identified sequences coverage and protein abundance were shown in the table.

f HEK293T cells stably expressing ZZ-Flag-SEC22B were harvested and subjected to ZZ and Flag IP, followed by MS to identify the SEC22B-binding proteins. Mass spectrometry analysis of ZZ-Flag-SEC22B binding proteins. The identified sequences coverage and protein abundance were shown in the table.

g Western blot analysis of STX18 knockout efficiency in STX18 KO HepG2 cells used in Fig. 1g.

h HepG2 cells were transfected with control or STX18 siRNA for 48 h and then mock treated or treated with Tunicamycin (Tun) or Thapsigargin (Tg) for 6 h. The cell lysates were analyzed via Western Blot.

i STX18 WT or KO U_2OS cells were treated with Tunicamycin (Tun), Thapsigargin (Tg) or Mock for 6 h. The cell lysates were assessed via RT-qPCR to measure the mRNA level of xbp1s. Error bars, mean ± SD of three independent experiments. Student's t test; * $P < 0.05$; NS, not significant.

j Western blot analysis of STX18 knockout efficiency of STX18 KO U_2OS cells used in Fig S1i.

k, l STX18 WT or KD HepG2 cells were treated with or without 0.2 mM OA-BSA for 6 h and then immune-stained with Sec31A (k) or Bap31 (l) antibodies and further

analyzed via immunofluorescence. Scale bar: 10 µm.

m HepG2 cells were transfected with control or STX18 siRNA. After 12 h, cells were transiently transfected with LiveDrop plasmid. 36 h later, cells were stained by hoechst for 10 minutes, and then applied to observation by confocal microscopy. Bars: 5 µm.

n Quantification of LiveDrop puncta area per cell (n=23 cells) in Supplementary Fig. S1m. The LiveDrop puncta area per cell was normalized to the average value of the control group cells. Unpaired t test. NS, not significant.

o Quantification of LiveDrop puncta number per cell (n=23 cells) in Supplementary Fig. S1m. Unpaired t test. NS, not significant.

Supplementary Fig. S2

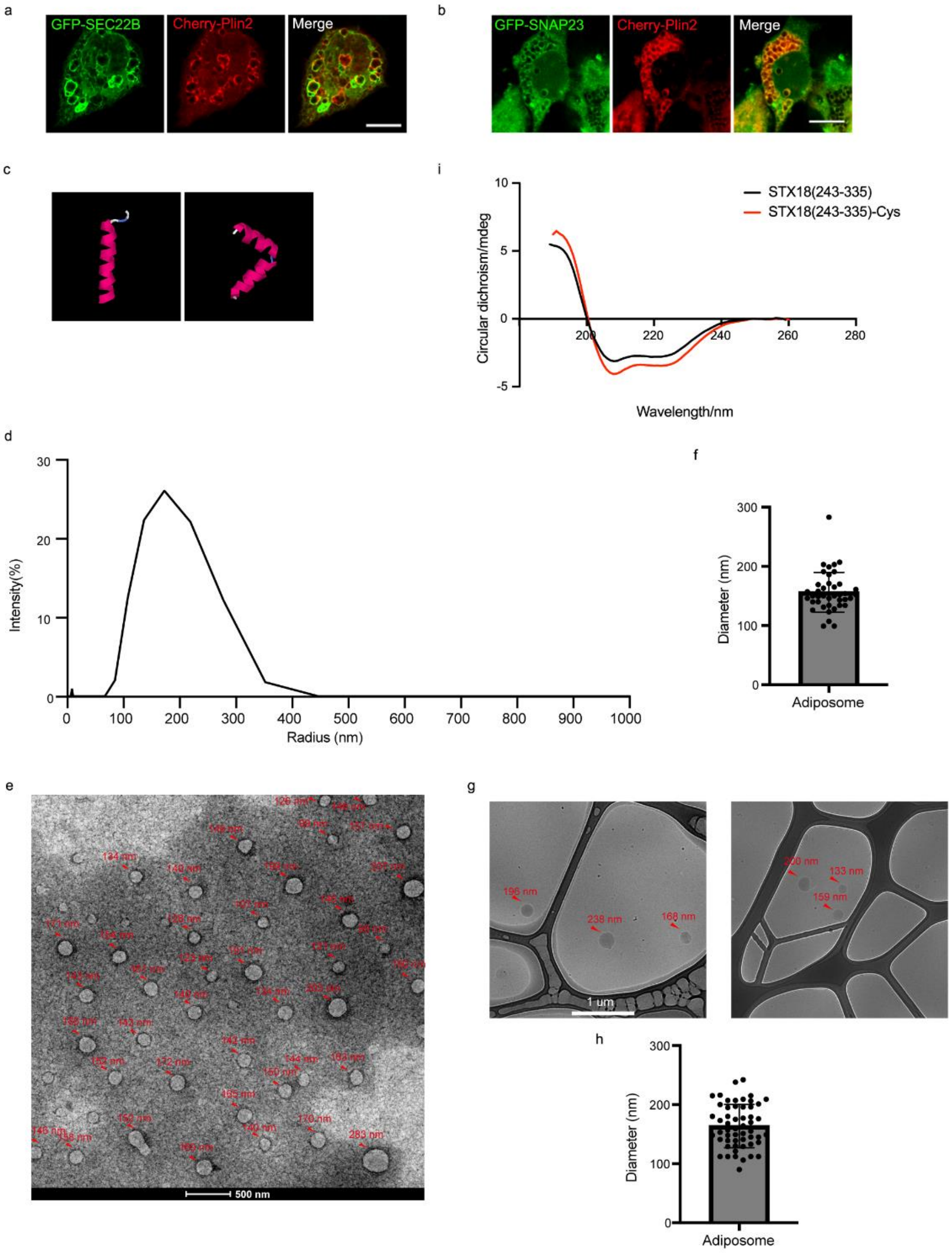

Supplementary Fig. S2: STX18, SNAP23 and SEC22B form a SNARE complex, locating on LDs.

a HepG2 cells were transfected with mCherry-Plin2 and GFP-SEC22B and then treated with or without 0.2 mM OA-BSA for 12 h, and cells were analyzed via fluorescence. Scale bar: 10 μm.

b HepG2 cells were transfected with mCherry-Plin2 and GFP-SNAP23 and then treated with or without 0.2 mM OA-BSA for 12 h, and cells were analyzed via fluorescence. Scale bar: 10 μm.

c The two possible structures of STX18-TMD (310-335) predicted by I-Tasser.

d The size of adiposomes was measured by DLS.

e Analysis of adiposome by ultra-thin transmission electron microscopy (TEM). Scale bar: 500 nm.

f Quantification of adiposomes size in Supplementary Fig. S2e. n=40 adiposomes.

g Analysis of adiposome by cryo-electron microscope. Scale bar: 1 μm.

h Quantification of adiposomes size in Supplementary Fig. S2g. n=54 adiposomes.

i The secondary structure information of STX18-SNARE-TMD and STX18-SNARE-TMD-Cys was obtained by circular dichroism.

Supplementary Fig. S3

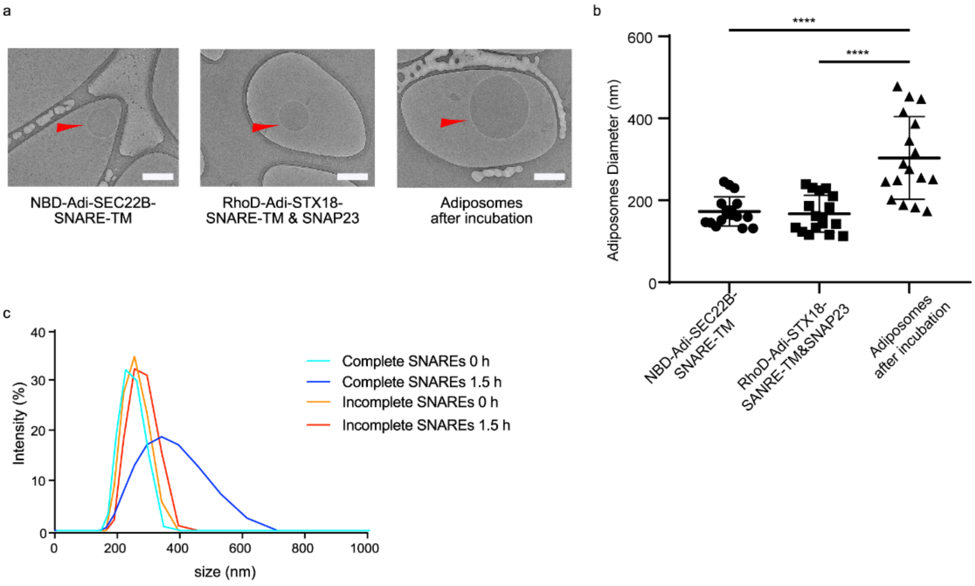

Supplementary Fig. S3: Assessment of adiposomes with SNAREs.

a Representative cryo-electron micrographs of adiposomes (n≥17) reconstituted with SNAREs. The NBD-Adiposomes were reconstituted with SEC22B-SNARE-TM, while the RhoD-Adiposomes were reconstituted with STX18-SNARE-TM and SNAP23. The two adiposomes were mixed with extra SNAP23 and incubated at 37°C for 2 h. The samples before and after incubation were applied to cyro EM for assessment. Scale bar: 200 nm.

b Quantification of adiposomes size (n≥17) in Supplementary Fig. S3a. Unpaired t test; **** P <0.0001

c Assessment of adiposomes size distribution by Dynamic light scattering (DLS). The NBD-Adiposomes were reconstituted with SEC22B-SNARE-TM, while the RhoD-Adiposomes were reconstituted with STX18-SNARE-TM and SNAP23. In the complete reaction set, the two adiposomes were mixed with extra SNAP23 and incubated at 37°C. For the incomplete reaction set, RhoD-Adi-STX18-SNARE-TM was used to replace RhoD-Adi-STX18-SNARE-TM&SNAP23 and no extra SNAP23 was included.

Supplementary Fig. S4

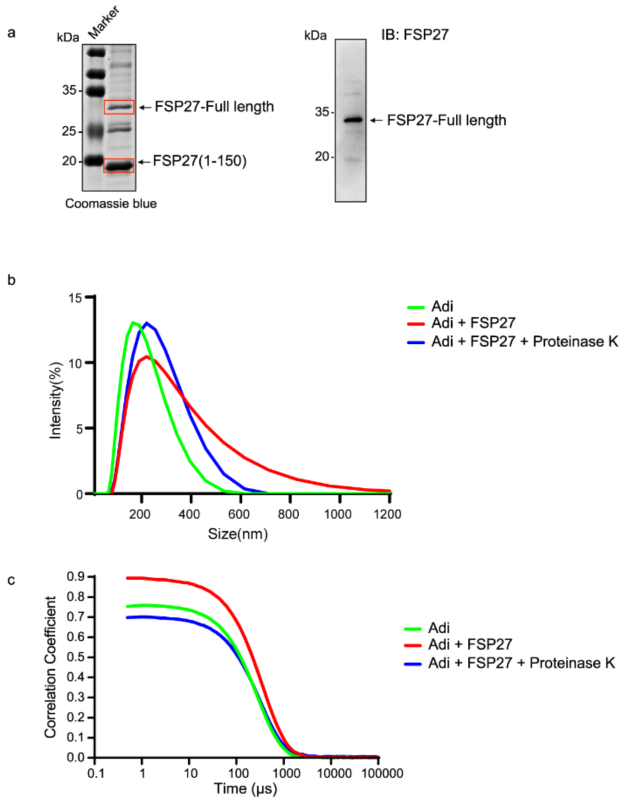

Supplementary Fig. S4: Purification of CIDEC/FSP27 and CIDEC/FSP27 (1-150).

a *E. Coli* purified CIDEC/FSP27 was subjected by SDS-PAGE (Coomassie blue staining) and Western blot against CIDEC/FSP27 antibody. The CIDEC/FSP27 (1-150) was identified by MS.

b, c Assessment of adiposomes size change by Dynamic light scattering (DLS) at 20°C on samples: Adiposomes alone, Adiposomes incubated with CIDEC/FSP27 for 2 h at 37°C, or Adiposomes incubated with CIDEC/FSP27 for 2 h first, and then with Proteinase K for another 2 h at 37°C. Data are presented as intensity vs size (b) or correlation coefficient vs time (c).

Synthetic procedures for DAG-CY3 and DAG-CY5

Unless otherwise noted, reagents and solvents were obtained from commercial suppliers. Reactions were monitored by thin-layer chromatography and were visualized with UV. Removal of solvents was conducted by using a rotary evaporator, and the residual solvent was removed from nonvolatile compounds using a vacuum manifold maintained at 1 torr. ^{1}H NMR and ^{13}C NMR spectra were obtained using a AVANCE NEO 400 (400 MHz) spectrometer (Bruker), and were analyzed using MestReNova 9.0 software. Chemical shifts are reported in parts per million (δ) relative to residual undeuterated solvent as an internal reference. Coupling constants (J) are reported in hertz. Spin multiplicities are described as s (singlet), brs (broad singlet), t (triplet), q (quartet), and m (multiplet). Mass spectra were recorded on LCMS2020 (Shimadzu). Mass spectra were obtained and analyzed using LabSolutions (Shimadzu).

Synthetic procedures for DAG-CY3

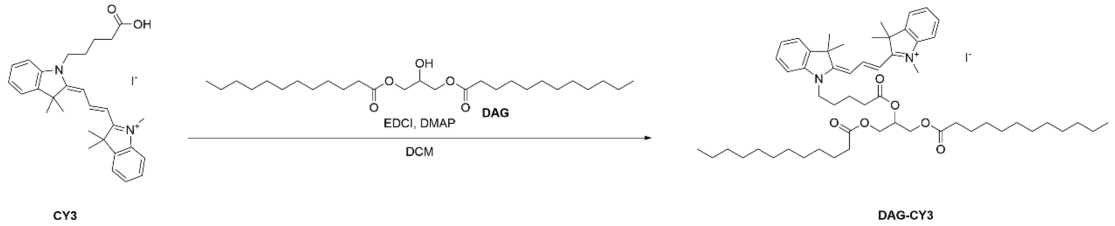

DAG-CY3

To a 25 mL round-bottomed flask was added CY3 (DuoFluor, D10122) (60 mg, 0.1 mmol) in DCM (2 mL). To this solution was added EDCI (19.7 mg, 0.1 mmol) and DMAP (2 mg, 0.02 mmol) and the reaction was stirred for 15 min. At this time, DAG (50 mg, 0.11 mmol) was added in DCM (2 mL) and the reaction was stirred for an additional 30 min. The solvent was then removed under reduced pressure, and the crude product purified by silica gel column chromatography (MeOH/DCM, 1/10) to give DAG-CY3 (20 mg) as a metallic blue solid.

^{1}H NMR (400 MHz, $CDCl_3$) δ 8.34 (s, 1H), 7.36 – 7.22 (m, 5H), 7.16 (d, J = 7.4 Hz, 1H), 7.06 (dd, J = 8.0, 4.5 Hz, 2H), 5.15 (t, J = 5.0 Hz, 1H), 4.21 (dd, J = 11.9, 4.4 Hz, 4H), 4.05 (dd, J = 11.9, 5.7 Hz, 2H), 3.73 (s, 3H), 2.32 (d, J = 7.4 Hz, 2H), 2.24 (t, J =

7.6 Hz, 4H), 1.81 (s, 2H), 1.19 (d, J = 10.1 Hz, 32H), 0.80 (t, J = 6.8 Hz, 6H).
LCMS found $[M]^+$ = 896.7

Synthetic procedures for DAG-CY5

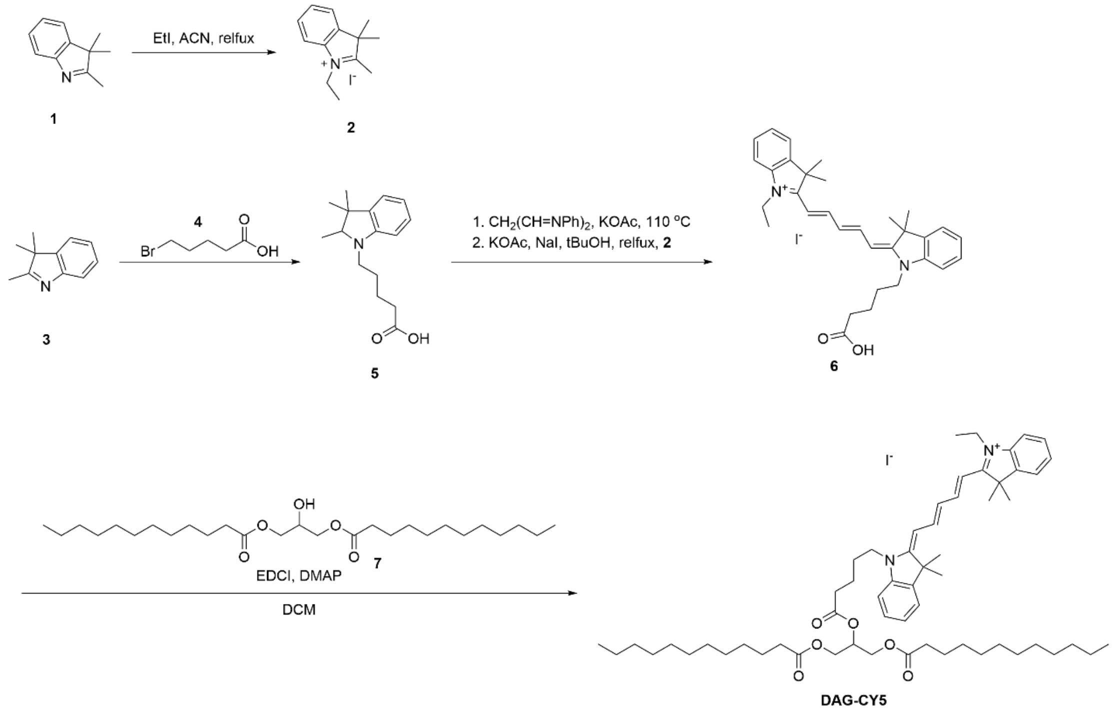

Compound 2

A solution compound 1 (7.95 g, 50 mmol), EtI (50 mmol) in ACN (50 mL) was heated at 110 ˚C for 24 h. The mixture was then cooled down to room temperature followed by the addition of 200 mL dry diethyl ether. The resulting product was isolated by filtration under reduced pressure, washed with ice-cold diethyl ether, and then dried under vacuum to give compound 2 (8.01 g) as off-white solid.

^{1}H NMR (400 MHz, DMSO) δ 8.16 – 7.91 (m, 1H), 7.88 – 7.82 (m, 1H), 7.66 – 7.60 (m, 2H), 4.50 (q, J = 7.3 Hz, 2H), 2.84 (s, 3H), 1.54 (s, 6H), 1.45 (t, J = 7.3 Hz, 3H).

Compound 5

To a 15 mL round-bottomed flask was added 5-bromovaleric acid (1.59 g, 10 mmol) and 2,3,3-trimethylindolenine (1.5 g, 8 mmol). The reaction was heated to 100 ºC with stirring for 3 h. The red solid was treated with 6 mL of EtOAc, heated at reflux for 2 min, and cooled allowing the solid product to settle at the bottom of the reaction vessel. The red supernatant was then removed from the solid and discarded. This solvent

treatment and removal was repeated once more with EtOAc and six times with acetone to give compound 5 (1.1 g) as light-pink solid.

^{1}H NMR (400 MHz, DMSO) δ 7.98 (dd, *J* = 6.5, 2.7 Hz, 1H), 7.85 (dd, *J* = 5.7, 2.9 Hz, 1H), 7.68 – 7.58 (m, 2H), 4.48 (t, *J* = 7.6 Hz, 2H), 2.84 (d, *J* = 3.0 Hz, 3H), 2.31 (t, *J* = 7.2 Hz, 2H), 1.87 (ddt, *J* = 15.2, 11.6, 5.7 Hz, 2H), 1.66 (q, *J* = 7.6 Hz, 2H), 1.54 (s, 6H).

Compound 6(CY5)

To a 25 mL round-bottomed flask was added malonaldehyde bis(phenylimine) monohydrochloride (466 mg, 1.93 mmol) and KOAc (183 mg, 2.0 mmol). To this solution was then added acetic anhydride (10 mL) and the mixture was heated to 110 ºC for 15 min. Compound 5 was then added in small aliquots over 30 min until the reaction had a light green color (100.0 mg, 0.32 mmol).Then compound 2 (121 mg, 0.4 mmol) was added and the reaction was stirred under reflux for 1.5 h. The reaction was concentrated and purified by silica gel column chromatography (MeOH/DCM, 1/10) to give compound 6(60 mg) as a metallic purple solid.

^{1}H NMR (400 MHz, MeOD) δ 8.28 (td, *J* = 13.1, 3.6 Hz, 2H), 7.52 (dd, *J* = 7.5, 4.1 Hz, 2H), 7.43 (dd, *J* = 8.1, 4.5 Hz, 2H), 7.32 (d, *J* = 8.3 Hz, 2H), 7.28 (dt, *J* = 7.4, 3.6 Hz, 2H), 6.66 (t, *J* = 12.4 Hz, 1H), 6.39 – 6.22 (m, 2H), 4.17 (dt, *J* = 16.7, 7.6 Hz, 4H), 2.39 (dt, *J* = 22.4, 7.0 Hz, 3H), 1.87 (d, *J* = 7.0 Hz, 3H), 1.82 – 1.70 (m, 13H), 1.41 (t, *J* = 7.1 Hz, 3H).

DAG-CY5

To a 25 mL round-bottomed flask was added CY6 (60 mg, 0.1 mmol) in DCM (2 mL). To this solution was added EDCI (19.7 mg, 0.1 mmol) and DMAP (2 mg, 0.02 mmol) and the reaction was stirred for 15 min. At this time, compound 7(DAG) (50 mg, 0.11 mmol) was added in DCM (2 mL) and the reaction was stirred for an additional 30 min. The solvent was then removed under reduced pressure, and the crude product purified by silica gel column chromatography (MeOH/DCM, 1/10) to give DAG-CY5(20 mg) as a metallic purple solid.

^{1}H NMR (400 MHz, $CDCl_3$) δ 8.10 (t, *J* = 12.9 Hz, 2H), 7.29 (t, *J* = 6.5 Hz, 4H), 7.16 (t, *J* = 6.8 Hz, 2H), 7.02 (dd, *J* = 8.1, 3.1 Hz, 2H), 6.87 (t, *J* = 12.4 Hz, 1H), 6.39 (dd, *J*

= 39.9, 13.5 Hz, 2H), 5.15 (p, J = 5.1 Hz, 1H), 4.24 (dd, J = 12.0, 4.4 Hz, 2H), 4.07 (tt, J = 12.0, 6.3 Hz, 6H), 2.38 (t, J = 6.1 Hz, 2H), 2.22 (t, J = 7.5 Hz, 4H), 1.70 (s, 12H), 1.52 (d, J = 7.7 Hz, 5H), 1.36 (t, J = 7.3 Hz, 3H), 1.18 (d, J = 4.7 Hz, 34H), 0.80 (t, J = 6.6 Hz, 7H).

LCMS found $[M]^+$ = 921.7
